# Objective clinical tests to inform decision‐making prior to return to sport in athletes with shoulder instability: A scoping review

**DOI:** 10.1002/ksa.70107

**Published:** 2025-10-27

**Authors:** Johan Högberg, Aleksandra Królikowska, Robert Prill, Alex Fails, Adam Popchak, Eric Hamrin Senorski

**Affiliations:** ^1^ Sportrehab Sports Medicine Clinic Gothenburg Sweden; ^2^ Sahlgrenska Sports Medicine Center Gothenburg Sweden; ^3^ Department of Health and Rehabilitation, Unit of Physiotherapy, Institute of Neuroscience and Physiology, Sahlgrenska Academy University of Gothenburg Gothenburg Sweden; ^4^ Physiotherapy Research Laboratory, University Centre of Physiotherapy and Rehabilitation, Faculty of Physiotherapy Wroclaw Medical University Wroclaw Poland; ^5^ Center of Orthopaedics and Traumatology, University Hospital Brandenburg/Havel Brandenburg Medical School Theodor Fontane Brandenburg a.d.H Germany; ^6^ Faculty of Health Sciences Brandenburg Brandenburg Medical School Theodor Fontane Brandenburg a.d.H. Germany; ^7^ Department of Physical Therapy, School of Health and Rehabilitation Sciences University of Pittsburgh Pittsburgh Pennsylvania USA

**Keywords:** bankart repair, isokinetic strength, isometric strength, latarjet, return to sport testing, shoulder assessment

## Abstract

**Purpose:**

This review systematically maps the existing research on objective return to sport criteria for shoulder injuries, with a focus on clinical tests that inform decision‐making.

**Methods:**

A scoping review was performed. The following databases were searched from inception up to July 2025: Cochrane Library, Embase, Medline, PEDRo, Cinahl and AMED. All studies, regardless of design, which assessed any kind of shoulder function with regard to return to sport decision‐making in individuals participating in sports and presented with a shoulder injury were included. The result was qualitatively presented in free text, tables and figures.

**Results:**

Eleven studies were identified that reported objective shoulder tests prior to return to sport in athletes after shoulder instability surgery. The tests evaluated various parameters, including range of motion, muscular strength, muscular endurance, power, plyometrics, movement quality and trunk control. The passing rates of isokinetic shoulder strength tests ranged from 40% to 70%, the isometric shoulder strength tests from 28% to 100%, for shoulder endurance tests from 70% to 81% and shoulder performance tests from 29% to 100%. These assessments were conducted within a time frame of 4–21 months following surgery. The rate of subsequent shoulder instability or re‐dislocation ranged from 5% to 10% after returning to sport.

**Conclusions:**

Passing rates for objective shoulder tests, including muscular strength and endurance, as well as performance tests, varied widely, with re‐dislocation rates between 5% and 10% after returning to sport. However, the predominance of case series limits the ability to draw definitive conclusions about the effectiveness of these tests in reducing the risk of subsequent shoulder instability.

**Level of Evidence:**

Level IV, scoping review of level III.

**Abbreviations**
ASH test: athletic shoulder test
ER: external rotation
ERET: external rotation endurance Test
IR: internal rotation
kg: kilograms
MeSH: medical subject headings
n: number
PRISMA‐ScR: the Preferred Reporting Items for Systematic Reviews and Meta‐Analysis Extension for Scoping Reviews
RoM: range of motion
RTS: return to sports
S: seconds
SD: standard deviation
SLAP: superior labrum anterior and posterior

## INTRODUCTION

A shoulder injury presents a significant challenge for individuals engaged in sports across various levels, exerting detrimental effects on performance, training and daily function [30]. The incidence of shoulder injuries in overhead sports ranges from 0.2 to 1.8 per 1000 h of play [2, 20, 36]. The rates of return to sport vary widely upon sustaining a shoulder injury, spanning from 20% to 90%, contingent upon the degree of shoulder demand inherent to the specific sport [19, 33]. Despite the relatively high incidence of shoulder injuries, there is a lack of high‐quality studies to inform clinicians regarding criteria for a safe return to sports [30].

Once athletes return to sport, the focus shifts to minimise the risk of re‐injury and addressing potential secondary complications that may arise when resuming sports. Re‐injury is a major concern, as the shoulder may not return to its pre‐injury state due to factors such as incomplete healing, improper loading during return to sport, or insufficient rehabilitation of specific muscle groups [38, 39]. Testing provides clinicians with standardised tools to objectively assess physical performance, strength, neuromuscular control and overall functional capacity following an injury or surgery. These assessments are essential in determining whether an athlete has regained sufficient physical ability to safely progress back to unrestricted athletic activities. Additionally, return to sport testing provides valuable insights into an athlete's rehabilitation progress, highlighting any remaining deficits that may require further strength development or neuromuscular re‐training. Such assessments help ensure that athletes do not return to sport prematurely, which could increase their risk of re‐injury or secondary complications. Although return to sport testing is widely implemented with the intent of reducing re‐injury risk within other fields, such as anterior cruciate ligament injuries [37], it is important to note that there is currently no prospective data confirming its effectiveness in preventing recurrent shoulder injuries [30]. Despite this, the systematic use of objective testing remains a key component in clinical decision‐making.

It is widely accepted that a criteria‐based approach is essential for determining an athlete's readiness to return to sport following a shoulder injury or surgery [30]. However, there is limited clarity regarding how this approach is implemented in practice and whether existing studies consistently utilise objective criteria to inform decision‐making [9, 10, 14]. This raises the question of how the current literature addresses the issue of return to sport decision‐making and whether such a standardised, criteria‐based framework is being effectively utilised in studies.

This scoping review aimed to systematically map the existing research on objective return to sport criteria for shoulder injuries, with a focus on clinical tests that inform decision‐making. The review sought to identify gaps in the current literature and offer insights into areas needing further investigation to enhance clinical practice and support informed decision‐making regarding the return to sport.

## METHODS

Given the limited number of studies and notable heterogeneity in the literature reporting return to sport criteria for athletes with shoulder injuries, a scoping review was chosen. The Preferred Reporting Items for Systematic Reviews and Meta‐Analysis Extension for Scoping Reviews (PRISMA‐ScR) checklist guided the development of this scoping review [34] (Supporting Information: Table 1).

### Eligibility criteria

Eligible studies for inclusion were based on the following:

(1) Study designs: Randomised controlled trials, longitudinal cohort studies, cross‐sectional studies, case‐control studies, case series and case studies written in English, Swedish, Danish or Norwegian. No restriction on publication date was applied.
(2) Population: Individuals participating in sports, irrespective of the type of sport. No restriction on age was applied.
(3) Shoulder injury: Presenting with a shoulder injury, irrespective of the type of injury.
(4) Criteria‐based return to sport assessment: Including range of motion, strength, endurance, power, or other functional tests. Studies had to assess any kind of shoulder function with regard to return to sport decision‐making to be included.

The following exclusion criteria were applied:

(1) Studies that assessed patient‐reported outcomes measures only without objective shoulder testing.
(2) Studies that did not specify how the objective shoulder testing was performed or did not provide a specific cut‐off to pass the test.
(3) Nerve injuries or pectoralis major tendon ruptures.
(4) Injuries primarily involving the elbow, for example, distal triceps or biceps brachii injuries.

### Information sources

A librarian at the Sahlgrenska University Hospital Library conducted a search of the following databases from inception up to July 2025: Cochrane Library, Embase, Medline, PEDRo, Cinahl and AMED. Additionally, the reference lists of the included studies and relevant systematic reviews were screened to enhance the search.

### Search strategy

The search included single words and combinations of words in the title and abstract and was supplemented with Medical Subject Headings (MeSH) terms. The search terms used were ‘return to sport’, ‘shoulder fractures’, ‘shoulder pain’, ‘shoulder injuries’, ‘shoulder joint’, ‘athlete, ‘sport’, ‘player’, ‘elite’, ‘exercise’, ‘exercise test’, ‘validity’, ‘reliability’, ‘isometric, ‘isokinetic, ‘overhead’, ‘rate of force development’, ‘biodex, ‘dynamometer’, ‘power’, ‘torque total peak’, ‘throwing’, ‘weak’, ‘pain’, ‘instability’, ‘stability’, ‘strength’, ‘stiffness’, ‘score’, ‘test’, ‘measures’ and ‘closed or open chain’. MeSH terms used were ‘return to sport’, ‘shoulder fractures’, ‘shoulder pain’, ‘shoulder injuries’, ‘shoulder joint’, ‘para‐athletes’, ‘athletes’, ‘sports’, ‘athletic injuries’ and ‘exercise test’.

A similar search strategy was used and adapted to the specific configuration of each respective database. A flow chart of the complete search is available as Supporting Information: Tables 2–5).

### Selection process

The study selection process was conducted using the Rayyan QCRI web application for systematic review [23]. Two authors (JH and AK) independently assessed the uploaded studies through a three‐step procedure. Initially, all studies were screened based on their titles to identify those potentially meeting the inclusion criteria. Subsequently, abstracts were reviewed, followed by a thorough examination of full‐text articles for studies meeting the inclusion criteria. A third author (EHS) examined the included studies and eventual studies in conflict.

### Data charting process

An Excel spreadsheet (version 16; Microsoft Corporation) was designed for data extraction, featuring the following categories: author, sub‐groups, year, title, journal, study design, purpose, conclusion, sample size, sex, age, body mass index, laterality, affected side, sport type, shoulder injury, type of surgery, shoulder test, test goal, test apparatus, cut‐off to pass the test, test duration, contraction type, contraction mode (e.g., isokinetic, isometric), test position, angular velocity, set, repetitions, set rest, time of assessment, test results, time at returning to sports, second injuries, time of second injuries. Data extraction was conducted by one author (J.H.) and uncertainties were resolved through consultation with the senior author (E.H.S.).

### Synthesis method

The studies identified for assessing objective shoulder tests prior to return to sport in athletes with shoulder injuries were compiled into tables. Study characteristics were described, including study design, year of publication, type of athletes, type of shoulder injury and surgery. Tests used in included studies to inform decision‐making were compiled in a table with description of the tests and used cut‐off criteria to pass the tests, with figures to illustrate the tests. Objective shoulder tests that did not specifically assess isolated muscle strength or endurance, but rather evaluated a combination of coordination, strength and/or power, were categorised as ‘performance’ tests. Lastly, the time of assessment, time to return to sport and rate of re‐injury were summarised in a table.

## RESULTS

### Selection of sources of evidence

**Figure 1:**
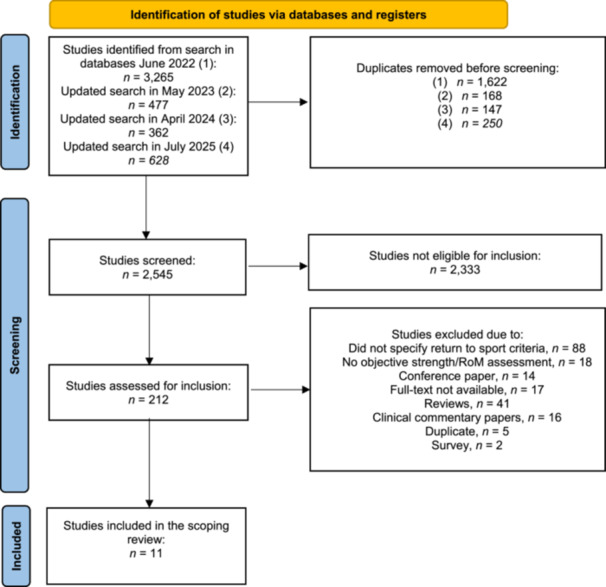
PRISMA flowchart of inclusion process. *n,* number; RoM, range of motion.

Four searches were performed, with the latest updated in July 2025. After the removal of duplicates, 2545 studies were screened, of which 212 were deemed eligible. After the application of exclusion criteria, eleven studies were included [4, 5, 16, 17, 25, 26, 27, 28, 31, 39, 40] (Figure 1).

### Characteristics of sources evidence

Of the eleven included studies, seven were case studies [5, 16, 25, 26, 31, 39, 40], one was a cross‐sectional study [17], one was a prospective cohort study [28], one was a retrospective cohort study [27] and one was a case‐control study [4]. In total, 557 athletes were included, with 72% of them being men. All studies included athletes with shoulder instability, with one study specifically investigating athletes who had revision surgery [31]. The most common sport athletes participated in was football. Further details of study characteristics are described in Table 1.

**Table 1:** Characteristics of included studies.

Authors	Study type	Sub‐groups	Surgery	No. of athletes, (% men)	Age, mean with SD (range)	Sports
Drummond Junior et al. [4]	Retrospective case–control	Criteria‐based RTS	Bankart repair	36 (83%)	20 (14–29)	Football *n* = 16 Basketball *n* = 3 Wrestling *n* = 2 Soccer *n* = 2 Ice hockey *n* = 2 Lacrosse *n* = 2 Other *n* = 9
		Control	Bankart repair	36 (64%)	19 (15–36)	Football *n* = 13 Basketball *n* = 3 Wrestling *n* = 4 Soccer *n* = 3 Ice Hockey *n* = 3 Lacrosse *n* = 1 Other *n* = 9
Eckenrode et al. [5]	Case series		Bankart repair	5 (100%)	20.2 (18–22)	Wrestling
Kelley et al. [16]	Case series		Bankart repair	62 (84%)	18.7 ± 2.0	Football *n* = 24 Ice hockey *n* = 24 Lacrosse *n* = 9 Basketball *n* = 4 Rugby *n* = 1
Khilfeh et al. 2025 [17]	Cross‐sectional		Anterior and/or posterior labral repair/capsulorrhaphy	59 (64%)	16.1 ± 1.7	Football *n* = 20 Other *n* = 8 Basketball *n* = 7 Soccer *n* = 6 Volleyball *n* = 4 Baseball *n* = 3 None *n* = 3 Dirt bike/motorcross *n* = 2 Lacrosse *n* = 2 Gymnastics *n* = 1 Martial Arts *n* = 1 Skiing/Snowboarding *n* = 1 Cheerleading *n* = 1
Pontillo et al. [25]	Case series		Anterior capsulabral repair (*n* = 1) Posterior capsulabral repair (*n* = 2) Combined (*n* = 2) SLAP (*n* = 1)	6 (100%)	18–21	Football
Reddy et al. [27]	Retrospective cohort	Criteria‐based RTS	Posterior labral repair (*n* = 16) Posterior labral repair and SLAP repair (*n* = 14)	30 (80%)	19.9 ± 4.2	Contact sports *n* = 16 Competitive level *n* = 25 Overhead sports *n* = 15
		Control	Posterior labral repair (*n* = 44) Posterior labral repair and SLAP repair (*n* = 23)	67 (69%)	22.5 ± 4.7	Contact sports *n* = 19 Competitive level *n* = 30 Overhead sports *n* = 30
Reddy et al. [26]	Case series		Latarjet	10 (80%)	19.9 ± 4.9	Contact sports *n* = 5 Overhead sports *n* = 6
Rogowski et al. [28]	Prospective cohort	Successful RTS	Open Latarjet	49 (88%)	22.6 ± 5.5	Collision *n* = 25 Contact *n* = 14 Overhead *n* = 7 Other *n* = 3
		No successful RTS		40 (93%)	25.7 ± 6.7	Collision *n* = 12 Contact *n* = 12 Overhead *n* = 11 Other *n* = 5
Sinha et al. [31]	Case series		Revision Bankart with remplissage	42 (62%)	28.2 ± 5.2	Non‐contact sports
Wilson et al. [39]	Case series		Anterior stabilisation surgery (*n* = 19) Posterior stabilisation surgery (*n* = 15) Anterior + posterior stabilisation surgery (*n* = 9)	43 (77%)	18.1 (15.1–21.8)	Football *n* = 24 Soccer *n* = 4 Baseball *n* = 6 Other *n* = 9
Yildiz et al. [40]	Case series		Anterior capsulabral repair	32 (100%)	24.5 ± 5.6	Type 2 *n* = 11* Type 3 *n* = 10** Type 4 *n* = 11***

*Note*: *Type 2: high‐impact sports, such as mixed martial arts, cycling, motorcross, soccer, rugby, water skiing, downhill skiing, parachuting and equestrianism. **Type 3: overhead sports, such as climbing, weightlifting, shot putting, crawl or butterfly swimming, pole vaulting, figure skating, canoeing, pitching, golf and field hockey. ***Type 4: overhead hitting movements such as basketball, handball, volleyball, goalkeeping, rugby, judo, karate or wrestling.

Abbreviations: *n*, number; RTS, return to sports; SD, standard deviation; SLAP, superior labrum anterior and posterior.

### Synthesis of employed shoulder tests

Among the eleven included studies, 18 different shoulder tests were identified as objective shoulder tests to inform decision‐making prior to return to sport. These tests evaluated range of motion, strength, endurance, power, plyometrics, trunk control and movement quality. Descriptions and interpretations of these tests are presented in Table 2.

**Table 2:** Description and interpretation of identified shoulder tests.

Test	What It measures	How to perform	Passing criteria
Range of motion	Shoulder flexibility (active and passive)	Assessed in flexion, abduction, internal and external rotation; performed actively and passively (supine)	≥90% of contralateral arm or preoperative values, except in cases of hypermobility [17, 25, 40]
Isokinetic strength (Figure 2)	Shoulder internal/external rotation strength	Seated; 45° shoulder abduction, 90° elbow flexion; five to six repetitions at 60°/s and 10 at 180°/s using an isokinetic dynamometer or seated with 90° shoulder abduction and 10° scaption.with either 180°/s or 300°/s	≥90% of contralateral arm [4, 17, 26, 27, 39, 40] and ≥70% external rotation:internal rotation ratio [17]
Isometric strength (Figures 3 and 4)	Shoulder internal/external rotation strength	Supine (0° shoulder abduction) or prone (90° shoulder abduction); 5‐s contraction, two trials with handheld dynamometer, 1–2 min rest in between	≥90% of contralateral arm [4, 5, 17, 26, 27, 28]
Posterior shoulder endurance test	Posterior rotator cuff and deltoid muscular endurance	Prone; 90° shoulder abduction, elbow extended; 2 kg dumbbell, lift from horizontal flexion to extension to failure	≥85% of contralateral arm [16]
External rotation endurance test (Figure 5)	External rotator endurance	Side‐lying (0° shoulder abduction) or prone (90° abduction), 90° elbow flexion; 5% body weight dumbbell to failure	≥90% of contralateral arm [4, 26, 27, 39]
Prone Y‐test	Shoulder muscular endurance	Prone; arm lifted to 120° shoulder abduction with full external rotation, parallel to floor; 3% body weight dumbbell, metronome paced (60 hertz)	≥90% of contralateral arm [25]
Scaption test	Shoulder muscular endurance	Standing; 90° shoulder elevation in scapular plane; 5% body weight dumbbell, metronome paced (60 hertz)	≥90% of contralateral arm [25]
Standing cable press test	Shoulder endurance and trunk control	Standing; press to 90° shoulder flexion with 30% body weight via cable to failure, metronome paced (60 hertz)	≥90% of contralateral arm [25]
CKC upper extremity stability test (Figure 6)	Shoulder strength and trunk control	Push‐up position; hands 91.4 cm apart; alternating taps as quickly as possible for 15 s	≥21 touches (average of three trials) [4, 16, 17, 25, 26, 27, 28, 40]
Unilateral shot‐put test (Figure 7)	Power and strength	Seated with back against a wall and knees bent; throw a 2.72 kg ball as far as possible, three trials/arm with 30‐s rest between throws	≥90% of contralateral arm (±10% for hand dominance) [4, 17, 26, 27, 28, 31, 39, 40]
One‐arm hop test	Plyometric power and trunk control	One‐arm push‐up stance; hop to 10.2 cm step‐up board five times as quickly as possible	Successful completion of five hops [16]
Overhand band reach test	Movement quality and scapular control	Standing; reach in I and Y motions with resistance band; avoid compensations	No compensatory movement [16]
Upper extremity Y balance test (Figure 8)	Dynamic shoulder stability, proprioception and trunk control	Push‐up position; reach in three directions; medial, superolateral, inferolateral for three trials for each direction; score compared to contralateral side	A composite score calculated for each direction; ≥90% of contralateral arm [16, 17, 28, 40]
Trunk control push‐up test	Trunk stability during upper body effort	Prone push‐up with controlled spine and hip movement; three repetitions	All three performed with control [16]
Long‐arm plank ball tap test	Shoulder endurance and stability and trunk control	Push‐up position; tap a medicine ball side‐to‐side 10 times	10 controlled taps [16]
Plank weight stacking test	Shoulder/trunk control under load	Push‐up position; move four 5‐kg plates side‐to‐side, four times	Completion of four repetitions with control [16]
Grip strength	Overall strength	Seated; neutral shoulder position with 90° elbow flexion	≥25th percentile of age and sex matched normative values [17]

Abbreviations: CKC, closed kinetic chain; cm, centimeters; kg, kilograms; s, seconds.

### Passing rates and second shoulder injuries

The timing of objective shoulder test assessments to return to sport ranged from 4 months [5] to 21 months [31] after surgery. Importantly, the objective shoulder test assessment at 21 months was after revision surgery [31]. Pontillo et al. [25] performed return to sport evaluations at 5–6 months postoperatively and then again at 8–10 months (prior to the start of the season). Two athletes failed to exceed 90% symmetry in the prone Y test, whereas the rest passed all tests. Nonetheless, all athletes returned to sport for the upcoming season.

Kelley et al. [16] reported that all athletes passed the objective shoulder testing at 6.19 ± 0.65 and returned to full competition at 6.50 ± 0.66 months after surgery.

Eckenrode et al. [5] did not specifically describe the time of the return to sport assessment, however, all athletes passed the isometric strength test and began training with the strength and conditioning coach at their wrestling team at 4 months and performed contact drills at 6 months after surgery. Four out of five athletes returned to wrestling, one of whom did not return due to school graduation.

Sinha et al. [31] recommended return to sport after 8 months and then return to competitive sports after 12 months, provided athletes had full confidence in their shoulder, were pain free and passed the seated shot‐put test. The average time to return to sport at the competitive level was 15.4 ± 1.4 months after surgery [31].

Yildiz et al. [40] performed objective shoulder testing 6 months after surgery. All patients recovered their passive range of motion compared to preoperative values. Limb symmetry for isokinetic external rotation strength ranged from 69% to 96% at 60°/s and from 64% to 79% at 180°/s. For isokinetic internal rotation strength, symmetry ranged from 89% to 96% at 60°/s and from 90% to 95% at 180°/s. Limb symmetry was 94.7% for the upper quarter Y balance test, 102.5% for the unilateral shot‐put test and the average score of the closed kinetic chain upper extremity stability test was 21.8 ± 2.6 touches [40].

The remaining studies performed return to sport test assessments between 5 and 6 months postoperatively [4, 17, 26, 27, 28, 39], and the results of these assessments are illustrated in Table 3. The time of returning to sports and the eventual occurrence of re‐injuries after returning to sport are described in Table 4.

**Table 3:** Pass rates for isokinetic shoulder muscle strength, isometric shoulder muscle strength, shoulder muscle endurance, the closed kinetic chain upper extremity stability test, the seated shot‐put test and the upper quarter Y balance test.

Test		Pass rate (≥90% of contralateral arm)
Isokinetic shoulder muscle strength	ER 60°/s	55% [27] 46% [4] 49% [39] 50% [26]
	ER 180°/s	66% [27] 46% [4] 44% [39] 40% [26]
	IR 60°/s	59% [27] 57% [4] 60% [39] 50% [26]
	IR 180°/s	69% [27] 57% [4] 70% [39] 40% [26]
Isometric shoulder muscle strength	ER 0°	90% [27] 60% [4] 60% [26]
	ER 90°	66% [27] 49% [4] 50% [26] 61% [17] 33% [28]
	IR 0°	90% [27] 62% [4] 70% [26]
	IR 90°	72% [27] 70% [4] 70% [26] 44% [17] 28% [28]
	ER/IR ratio at 90°	89% [28]
Shoulder muscular endurance	ERET 0°	78% [27] 73% [4] 72% [39] 80% [26]
	ERET 90°	73% [27] 81% [4] 67% [39] 70% [26]
The CKC upper extremity stability test		90% [27] 70% [4] 77% [39] 90% [26] 60% [28]
The unilateral shot‐put test		93% [27] 87% [4] 79% [39] 90% [26] 29% [28]
The upper quarter Y balance test		59% [17] 81% [28]

Abbreviations: CKC, closed kinetic chain; ER, external rotation; ERET, external rotation endurance test; IR, internal rotation.

**Table 4:** Time until clearance for return to sport after surgery and eventual occurrence of re‐injuries.

Authors	Sub‐groups	Time until clearance for RTS after surgery (months)	Subsequent injury/issues	Time to re‐injury after surgery (months)	Follow‐up time of the included studies
Kelley et al. [16]		6.19 ± 0.65 (5–9)	Redislocation (6.5%)	–	2 years
Reddy et al. [27]	Criteria‐based RTS*	6.5	Recurrent instability (6.7%)	–	32.1 ± 17.2 months
	Control**	6.6	Recurrent instability (9.0%)	–	38.6 ± 24.7 months
Drummond Junior et al. [4]	Criteria‐based RTS	6–8	Recurrent instability (5.0%)	12	Minimum 1‐year follow‐up
	Control	5–6	Recurrent instability (22.0%)	13.6	Minimum 1‐year follow‐up
Pontillo et al. [25]		5–6 and 8–10	No injury	–	6 years
Eckenrode et al. [5]		6	No injury	–	One season after surgery
Sinha et al. [31]***		15.4 ± 1.4 (13–21)	Recurrent instability (9.5%) Persisting pain (2.4%)	–	
Reddy et al. 2024 [26]		6.4 ± 1.8	Recurrent instability (10%)	–	3.6 ± 1.2 years

Abbreviation: RTS, return to sports.

* RTS outcomes available for 19 athletes.

** RTS outcomes available for 35 athletes.

*** Athletes with revision surgery only.

**Figure 2:**
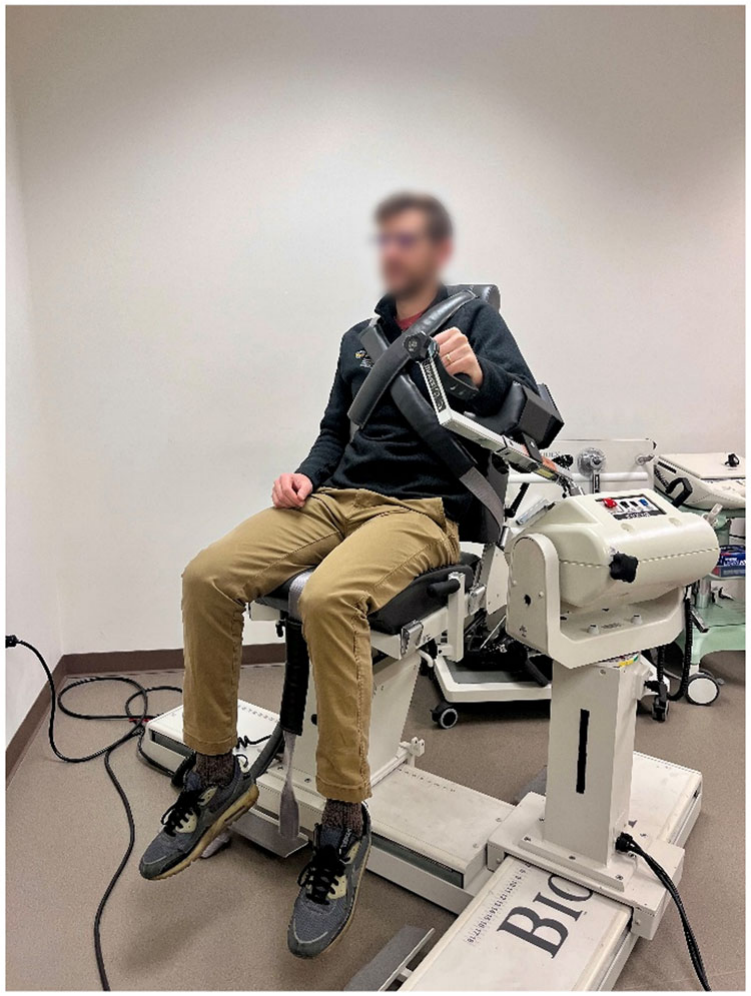
Isokinetic external and internal rotation shoulder strength testing in a Biodex.

**Figure 3:**
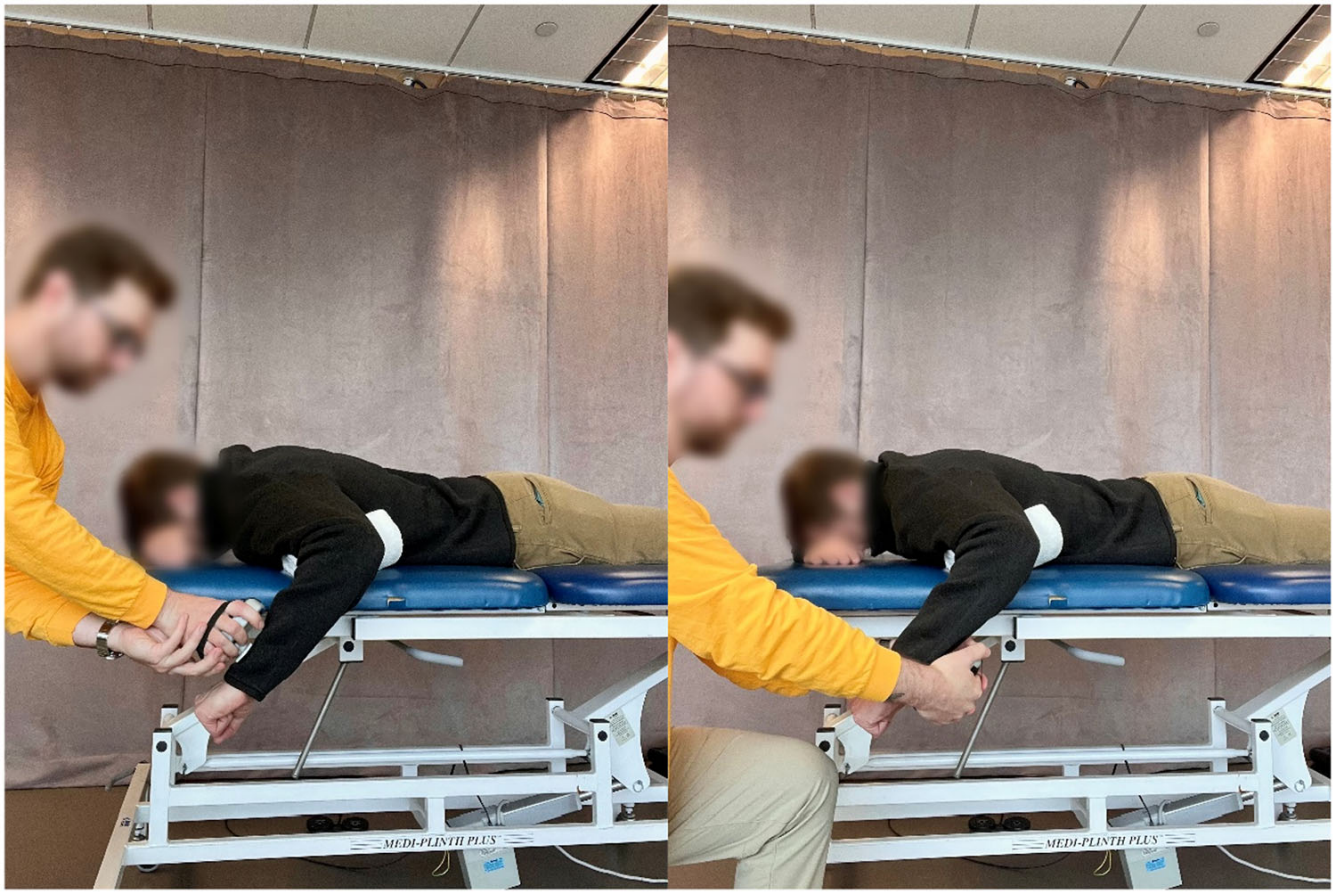
Isometric external (left picture) and internal (right picture) rotation shoulder strength testing in 90° of shoulder abduction.

**Figure 4:**
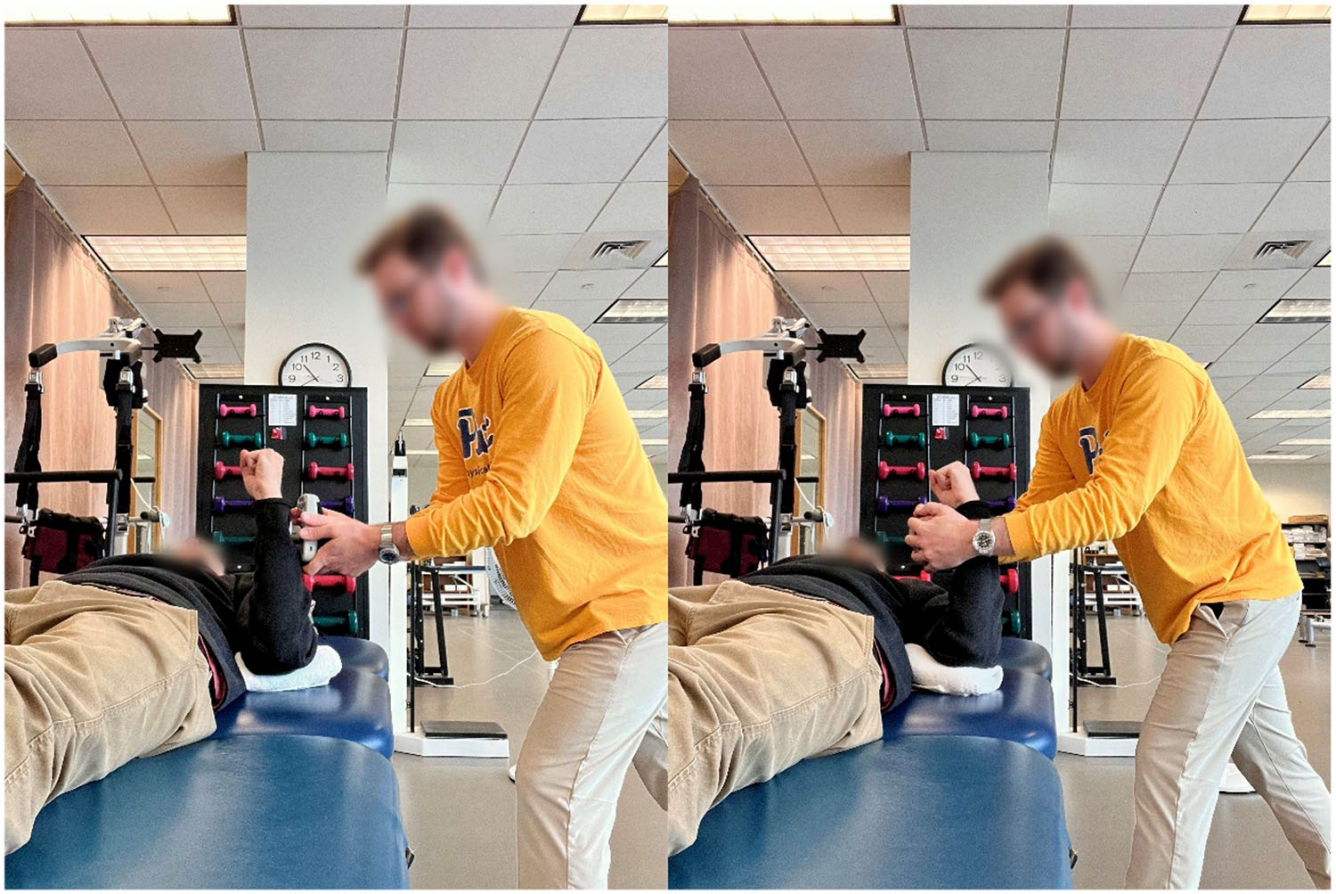
Isometric external (left picture) and internal (right picture) rotation shoulder testing in 0° of shoulder abduction.

**Figure 5:**
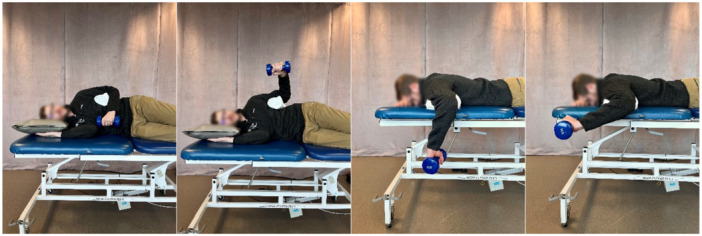
External rotation endurance tests in (1) side‐lying with 0° of shoulder abduction (to the left) and (2) 90° of shoulder abduction (to the right).

**Figure 6:**
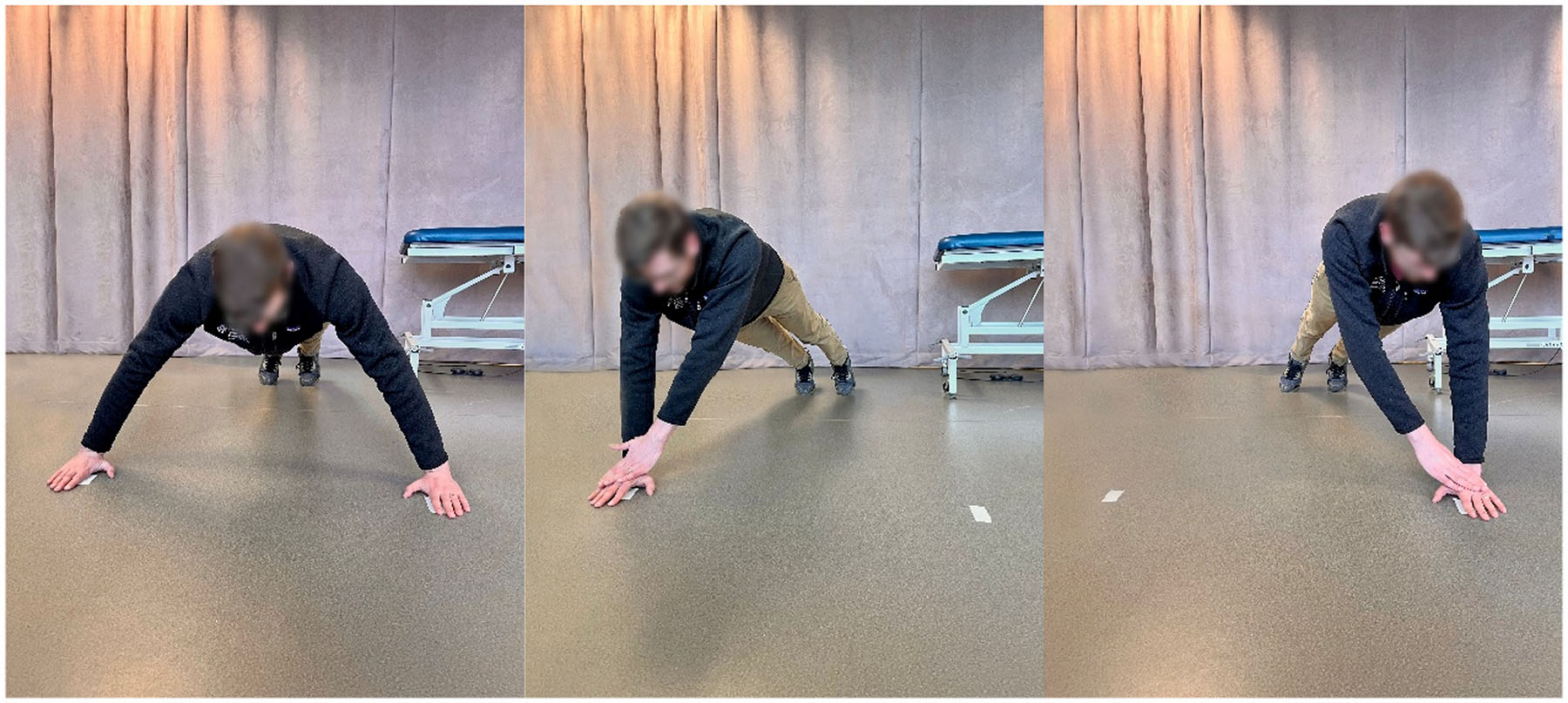
The closed kinetic chain upper extremity stability test.

**Figure 7:**
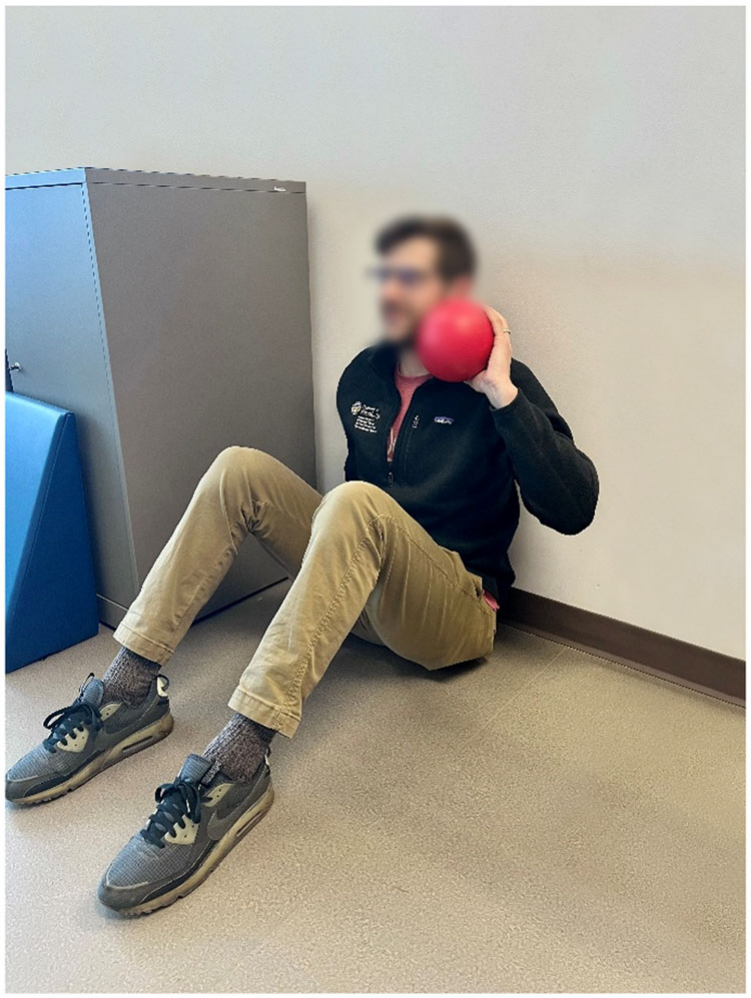
The seated shot‐put test.

**Figure 8:**
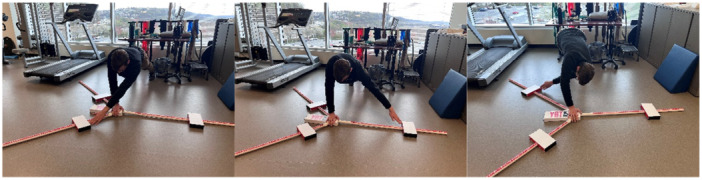
The upper extremity Y balance test. From left to the right: (1) superolateral direction, (2) medial direction and (3) inferolateral direction.

## DISCUSSION

### Summary of evidence

The most important findings of this scoping review were that a limited number of studies have reported on objective shoulder tests used to inform decision‐making prior to return to sport in athletes with shoulder instability. The included studies evaluated a wide range of shoulder function parameters—including strength, endurance, range of motion, power, plyometrics, movement quality and trunk control—but with considerable variation in the types of tests used. The reported passing rates varied widely, and the majority of studies were case series, limiting the evidence supporting any specific objective return to sport criteria test battery to reduce subsequent shoulder instability/injury. The diversity in clinical tests highlights a key gap in current objective shoulder tests and criteria for passing to inform decision‐making prior to return to sport. Clinically, this scoping review underscores the need for a more standardised, evidence‐based battery with clinical tests and objective return to sport criteria to inform decision‐making that go beyond time‐based milestones. A comprehensive battery of clinical shoulder tests may better capture the physical shoulder readiness and potentially reduce the risk of subsequent shoulder injury/instability upon returning to sport. However, future studies with prospective cohorts and controlled trials are needed to validate these tests and determine their utility in guiding return to sport decisions.

### Objective return to sport criteria

A consensus statement was published in 2022 [30] delineating six pivotal domains for consideration in the decision‐making process regarding return to sport: (1) pain, (2) active range of motion in the shoulder joint, (3) physical capacity encompassing strength, power and endurance, (4) the kinetic chain, (5) psychological factors and (6) sport‐specific activities. Given the scarcity of research specifically addressing return to sport protocols for shoulder injuries, the consensus statement predominantly relied on expert opinions. Objective return to sport criteria are generally considered to facilitate the safe return of athletes to sports with minimal risk of a second shoulder injury. However, Stone et al. [32] reported that 62% of studies examining the return to sport rate after open Bankart repair did not specify criteria for return to sport. Among those that did specify criteria, time‐based measures were the most common, with non‐contact athletes returning between 12 and 16 weeks, and athletes involved in throwing/contact returning by 6 months [32]. A minority (12%) of studies in Stone et al. [32] indicated that the shoulder strength should be at least 75%–80% of the uninjured side, without detailing how the strength should be measured. Fried et al. [7] similarly reported that only 60% of studies examining return to sport after arthroscopic stabilisation for posterior shoulder instability provided specific criteria. Despite this, the time‐based criteria remain the most commonly used approach following various types of surgery for shoulder instability [3, 10, 15, 32], with 6 months being the most common time frame.

Although time is an important factor in ensuring adequate healing of various shoulder structures, a purely time‐based criterion may risk permitting athletes to return to sports before full recovery. Wilson et al. [39] reported that only seven athletes (16%) passed all isokinetic strength tests 6 months after surgery. In line, Drummond Junior et al. [4] reported that six athletes (16%) passed all isokinetic strength tests 6 months after surgery. Among the six studies [4, 17, 26, 27, 39, 40] which assessed the isokinetic shoulder strength, the passing rate test ranged from 40% to 70%. These results highlight the importance of objective shoulder testing and not relying on time‐based criterion alone. However, Hargreaves et al. [11] reported in a cohort of healthy volunteers that no individual passed all tests in a criteria‐based return to sport protocol developed for athletes after Bankart repair. Interestingly, the non‐dominant arm had a deficit in 4 of the 12 bilateral arm tests [11], which questions the use of symmetry between the injured and the non‐injured limb without taking the dominant arm into account. Rogowski et al. [28] also investigated the symmetry between the dominant to non‐dominant limb in addition to the comparison of the injured versus the non‐injured limb. They reported that the recovery of dominant to non‐dominant limb symmetry in maximal internal rotation shoulder strength and shoulder muscle endurance at 4.5 months were predictors for return to sport at 1 year following anterior shoulder stabilisation via the open Latarjet procedure. Despite this, a majority of physical therapists do not incorporate objective shoulder tests when determining return to sport for athletes with upper extremity injuries [8]. This is worrying, especially as perceived physical shoulder deficits have been associated with failure to return to sport after arthroscopic Bankart repair [13]. Our results underline the importance of evaluating shoulder strength and performance using discrete, sensitive and objective measurements prior to returning to sport.

### Subsequent shoulder injuries

The rate of subsequent shoulder instability or redislocation in the included studies ranged from 5% to 10%. In the literature, the overall rate of recurrent instability following Bankart repair has been reported as 31.2%, with 16.0% sustaining recurrent dislocations [22]. Additionally, patients undergoing Bankart repair have a 3.08 times higher risk of recurrence and revision compared to those undergoing the Latarjet procedure [12].

Most of the studies included in this review had a case series design, which limits the ability to draw conclusions whether objective shoulder tests to inform decision‐making prior to return to sport may reduce the recurrence rate. However, two studies included a control group [4, 27]. Drummond Junior et al. [4] reported that athletes who followed a criteria‐based return to sport testing protocol after arthroscopic Bankart repair had a lower recurrence rate of instability compared to those who did not (5.0% vs. 22.0%). In contrast, Reddy et al. [27], did not observe a difference in recurrence between athletes who received time‐based clearance and those who underwent return to sport testing following primary arthroscopic posterior labral repair. The cohort in Reddy et al. [27] was on arthroscopic posterior labral repair, whereas the cohort in Drummond Junior et al. [4] was on primarily anterior labral repairs. The discrepancy in the type of shoulder instability between these two studies [4, 27] might have influenced the reported results. Whether the type of shoulder instability influences the sensitivity of the test battery needs further investigation. Collectively, the impact of objective shoulder tests to inform decision‐making prior to return to sport on recurrence rates remains inconclusive. On the other hand, these criteria effectively identify shoulder deficits, which may be important for informing decision‐making for returning to sport.

### Missing pieces and future directions in the objective return to sport criteria

The tests included in a objective shoulder test battery prior to return to sport typically evaluate the strength, endurance, mobility, performance and movement quality of the shoulder. However, the most cited reasons for not returning to sport are recurrent or persistent instability and/or fear of reinjury [18, 35]. Pasqualini et al. [24] observed that athletes who experienced recurrent shoulder instability were less psychologically prepared to return to sport, as assessed by the shoulder instability‐return to sport after injury scale, compared to those who did not experience a recurrence (20% vs. 4.3%). Therefore, in addition to evaluating strength, endurance, mobility, performance and movement quality, it is essential to consider psychological preparedness to return to sport and the athlete's subjective sense of shoulder stability.

When designing an objective shoulder test battery to inform decision‐making prior to return to sport, the injury mechanism should be considered. For patients with anterior shoulder dislocations, the most common mechanism of anterior shoulder dislocation involves a fall or direct contact to the upper extremity, often from an opponent [21, 29]. These injuries typically occur rapidly and often involve a long‐lever force. A test designed to replicate the stresses of a long‐lever moment and the ability to transfer force across the shoulder girdle at high velocity and load is the Athletic Shoulder (ASH) test [1]. This test measures maximum force and rate of force development while the athlete is in a prone position with the shoulder at three different angles: 180°, 135° and 90°. Edwards et al. [6] highlighted significant interlimb asymmetries at 4–6 months after surgery in the three different angles for muscle force and rate of force development at 200 ms in the ASH test. Although, the differences with a healthy control group demonstrated greater effect sizes than the comparison with the healthy limb, which suggests that the comparison with the non‐injured limb may be misguiding. Nonetheless, incorporating a test that assesses the muscle force and rate of force development in a long‐lever position could provide valuable insights in addition to the strength, endurance, mobility, performance and movement quality tests currently used.

### Limitations

The included studies focused exclusively on athletes with shoulder instability. Therefore, the findings may not apply to other types of shoulder injuries. The inclusion of only athletes with shoulder instability may be considered as a strength, however, the various types of instability and treatments are a limitation. There was considerable variability in the sports represented, and different sports impose distinct demands on the shoulder. It remains debated whether return to sport test batteries should be tailored to specific sports. For example, contact sports may benefit from more closed kinetic chain tests, while overhead sports involving throwing may require more open kinetic chain tests to better reflect sport‐specific needs. One universal test battery may not be suitable for all athletes or all types of shoulder instability. Additionally, many studies had small sample sizes, and most lacked a control group, limiting the ability to draw definitive conclusions about whether an objective criteria‐based shoulder test battery prior to return to sport reduces the risk of shoulder instability recurrence. Future research should explore whether passing an objective criteria‐based shoulder test battery prior to return to sport decreases the risk of recurrence compared to athletes who do not pass, grouped for the type of shoulder instability.

## CONCLUSION

Passing rates for objective shoulder tests, including muscular strength and endurance, as well as performance tests, varied widely, with redislocation rates between 5% and 10% after returning to sport. However, the predominance of case series limits the ability to draw definitive conclusions about the effectiveness of these tests in reducing the risk of subsequent instability.

## AUTHOR CONTRIBUTIONS

Johan Högberg drafted the initial version of the manuscript. Johan Högberg, Aleksandra Królikowska, Robert Prill, and Eric Hamrin Senorski contributed substantially to the acquisition of the data and the analysis of the data, and they are responsible for drafting the manuscript and revising it critically for important intellectual content. Alex Fails and Adam Popchak made large contributions to the revision and design of the work. Johan Högberg and Eric Hamrin Senorski are responsible for the concept of design.

## CONFLICTS OF INTEREST STATEMENT

The authors declare no conflicts of interest.

## ETHICS STATEMENT

The authors have nothing to report.

## Supporting information

Supplementary Tables.

## Data Availability

Data are available on reasonable request.

## References

[1] Ashworth B , Hogben P , Singh N , Tulloch L , Cohen DD . The Athletic Shoulder (ASH) test: reliability of a novel upper body isometric strength test in elite rugby players. BMJ Open Sport Exerc Med. 2018;4(1):e000365. 10.1136/bmjsem-2018-000365 PMC6059329 30057775

[2] Bahr R , Reeser JC . Injuries among world‐class professional beach volleyball players. The Fédération Internationale de Volleyball beach volleyball injury study. Am J Sports Med. 2003;31(1):119–125. 12531768 10.1177/03635465030310010401

[3] Ciccotti MC , Syed U , Hoffman R , Abboud JA , Ciccotti MG , Freedman KB . Return to play criteria following surgical stabilization for traumatic anterior shoulder instability: a systematic review. Arthroscopy. 2018;34(3):903–913. 29146162 10.1016/j.arthro.2017.08.293

[4] Drummond Junior M , Popchak A , Wilson K , Kane G , Lin A . Criteria‐based return‐to‐sport testing is associated with lower recurrence rates following arthroscopic Bankart repair. J Shoulder Elbow Surg. 2021;30(7s):S14–S20. 33798726 10.1016/j.jse.2021.03.141

[5] Eckenrode BJ , Logerstedt DS , Sennett BJ . Rehabilitation and functional outcomes in collegiate wrestlers following a posterior shoulder stabilization procedure. J Orthop Sports Phys Ther. 2009;39(7):550–559. 19574657 10.2519/jospt.2009.2952

[6] Edwards PK , Blackah N , McEwan J , D'Alessandro P , Ebert JR . Deficits in upper limb long lever isometric force after shoulder stabilization surgery in Australian Rules Footballers. Orthop J Sports Med. 2025;13(7):23259671251342585. 40620724 10.1177/23259671251342585 PMC12227849

[7] Fried JW , Hurley ET , Duenes ML , Manjunath AK , Virk M , Gonzalez‐Lomas G , et al. Return to play after arthroscopic stabilization for posterior shoulder instability: a systematic review. Arthrosc Sports Med Rehabil. 2021;3(1):e249–e256. 33615272 10.1016/j.asmr.2020.08.007 PMC7879176

[8] Gauthier ML , Unverzagt CA , Mendonça LDM , Seitz AL . Missing the forest for the trees: a lack of upper extremity physical performance testing in sports physical therapy. Int J Sports Phys Ther. 2023;18(2):419–430. 37020447 10.26603/001c.73791 PMC10069373

[9] Gofflot A , Croisier JL , Kaux JF , Delvaux F , Tubez F , Tooth C , et al. Return to play decision after shoulder dislocation in upper limb athletes: critical analysis between the habits of medical professionals and the literature. Orthop Traumatol Surg Res. 2024;110(1):103715. 37865233 10.1016/j.otsr.2023.103715

[10] griffith r , fretes n , bolia ik , murray ir , meyer j , weber ae , et al. return‐to‐sport criteria after upper extremity surgery in athletes: a scoping review, part 1: rotator cuff and shoulder stabilization procedures. Orthop J Sports Med. 2021;9(8):23259671211021827. 34395687 10.1177/23259671211021827 PMC8358521

[11] Hargreaves M , Wood A , Manfredi N , Dayal D , Hudson J , Pyrz KH , et al. A high percentage of healthy volunteers fail to pass criteria‐based return to sport testing for arthroscopic Bankart repair. Arthroscopy. 2025;41(8):2785–2791. 39914614 10.1016/j.arthro.2025.01.047

[12] Hossein Zadeh R , Daliri M , Sadeghi M , Hossein Zadeh R , Sahebi M , Moradi A , et al. Arthroscopic Bankart repair vs. Latarjet procedure for recurrent shoulder instability: a meta‐analysis of clinical outcomes and complication rates in general and athletic populations. J Shoulder Elbow Surg. 2024;33(12):e652–e674. 39151667 10.1016/j.jse.2024.06.024

[13] Hurley ET , Davey MS , Mojica ES , Montgomery C , Gaafar M , Jazrawi LM , et al. Analysis of patients unable to return to play following arthroscopic Bankart repair. Surgeon. 2022;20(4):e158–e162. 34366225 10.1016/j.surge.2021.06.005

[14] Hurley ET , Matache BA , Colasanti CA , Mojica ES , Manjunath AK , Campbell KA , et al. Return to play criteria among shoulder surgeons following shoulder stabilization. J Shoulder Elbow Surg. 2021;30(6):e317–e321. 33618019 10.1016/j.jse.2021.01.026

[15] Hurley ET , Montgomery C , Jamal MS , Shimozono Y , Ali Z , Pauzenberger L , et al. Return to play after the Latarjet procedure for anterior shoulder instability: a systematic review. Am J Sports Med. 2019;47(12):3002–3008. 31038983 10.1177/0363546519831005

[16] Kelley TD , Clegg S , Rodenhouse P , Hinz J , Busconi BD . Functional rehabilitation and return to play after arthroscopic surgical stabilization for anterior shoulder instability. Sports Health. 2022;14(5):733–739. 34918564 10.1177/19417381211062852 PMC9460095

[17] Khilfeh B , Wong C , Gupta A , Saper M . Return to sport testing after arthroscopic shoulder surgery in adolescent and young adult patients. Orthop J Sports Med. 2025;13(7):23259671251352191. 40656655 10.1177/23259671251352191 PMC12254610

[18] Kim M , Haratian A , Fathi A , Kim DR , Patel N , Bolia IK , et al. Can we identify why athletes fail to return to sports after arthroscopic bankart repair? A systematic review and meta‐analysis. Am J Sports Med. 2022;51(9):2480–2486. 35658631 10.1177/03635465221089980

[19] Michener LA , Abrams JS , Bliven KCH , Falsone S , Laudner KG , McFarland EG , et al. National Athletic Trainers’ Association Position Statement: evaluation, management, and outcomes of and return‐to‐ play criteria for overhead athletes with superior labral anterior‐posterior injuries. J Athl Train. 2018;53(3):209–229. 29624450 10.4085/1062-6050-59-16 PMC5894372

[20] Moller M , Attermann J , Myklebust G , Wedderkopp N . Injury risk in Danish youth and senior elite handball using a new SMS text messages approach. Br J Sports Med. 2012;46(7):531–537. 22554848 10.1136/bjsports-2012-091022

[21] Montgomery C , O'Briain DE , Hurley ET , Pauzenberger L , Mullett H , Moran CJ . Video analysis of shoulder dislocations in rugby: insights into the dislocating mechanisms. Am J Sports Med. 2019;47(14):3469–3475. 31710508 10.1177/0363546519882412

[22] Murphy AI , Hurley ET , Hurley DJ , Pauzenberger L , Mullett H . Long‐term outcomes of the arthroscopic Bankart repair: a systematic review of studies at 10‐year follow‐up. J Shoulder Elbow Surg. 2019;28(11):2084–2089. 31311748 10.1016/j.jse.2019.04.057

[23] Ouzzani M , Hammady H , Fedorowicz Z , Elmagarmid A . Rayyan: a web and mobile app for systematic reviews. Syst Rev. 2016;5(1):210. 27919275 10.1186/s13643-016-0384-4 PMC5139140

[24] Pasqualini I , Rossi LA , Hurley ET , Turan O , Tanoira I , Ranalletta M . Shoulder instability‐return to sports after injury scale shows that lack of psychological readiness predicts outcomes and recurrence following surgical stabilization. Arthroscopy. 2024;40(12):2815–2824. 38735414 10.1016/j.arthro.2024.04.030

[25] Pontillo M , Sennett BJ , Bellm E . Use of an upper extremity functional testing algorithm to determine return to play readiness in collegiate football players: a case series. Int J Sports Phys Ther. 2020;15(6):1141–1150. 33344031 10.26603/ijspt20201141 PMC7727439

[26] Reddy RP , Como M , Charles S , Herman ZJ , Nazzal EM , Como CJ , et al. Criteria‐based return to sport testing after open Latarjet reveals residual deficits and can be utilized for sports clearance with excellent outcomes at mean 3.6 year follow‐up: a small case series of competitive athletes. Phys Ther Sport. 2024;65:23–29. 37995416 10.1016/j.ptsp.2023.11.002

[27] Reddy RP , Rai A , Como M , Sebastiani R , Como C , Hyre N , et al. Criteria‐based return‐to‐sport testing helps identify functional deficits in young athletes following posterior labral repair but may not reduce recurrence or increase return to play. JSES Int. 2023;7(3):385–392. 37266173 10.1016/j.jseint.2023.01.002 PMC10229405

[28] Rogowski I , Nové‐Josserand L , Godenèche A , Collotte P , Franger G , Borel F , et al. Are the dominant‐nondominant functional differences at 4.5 months after open Latarjet procedure better predictors for successful return to sport at 1 year postoperatively than the operated‐nonoperated differences? J Shoulder Elbow Surg. 2025;34:2338–2349. 39954984 10.1016/j.jse.2024.12.046

[29] Schneider KN , Schachtrup T , Gosheger G , Hiort ML , Degener BJ , Zafeiris T , et al. Systematic video analysis of shoulder dislocations in professional male football (soccer): injury mechanisms, situational and kinematic patterns. J Exp Orthop. 2024;11(4):e70121. 39703833 10.1002/jeo2.70121 PMC11656221

[30] Schwank A , Blazey P , Asker M , Møller M , Hägglund M , Gard S , et al. 2022 bern consensus statement on shoulder injury prevention, rehabilitation, and return to sport for athletes at all participation levels. J Orthop Sports Phys Ther. 2022;52(1):11–28. 34972489 10.2519/jospt.2022.10952

[31] Sinha S , Mehta N , Goyal R , Goyal A , Joshi D , Arya RK . Is revision Bankart repair with remplissage a viable option for failed bankart repair in non‐contact sports person aiming to return to sports? Indian J Orthop. 2021;55(Suppl 2):359–365. 34306548 10.1007/s43465-021-00415-4 PMC8275742

[32] Stone GP , Pearsall AW . Return to play after open bankart repair: a systematic review. Orthop J Sports Med. 2014;2(2):2325967114522960. 26535302 10.1177/2325967114522960 PMC4555624

[33] Thorsness R , Alland JA , McCulloch CB , Romeo A . Return to play after shoulder surgery in throwers. Clin Sports Med. 2016;35(4):563–575. 27543399 10.1016/j.csm.2016.05.003

[34] Tricco AC , Lillie E , Zarin W , O'Brien KK , Colquhoun H , Levac D , et al. PRISMA Extension for Scoping Reviews (PRISMA‐ScR): checklist and explanation. Ann Intern Med. 2018;169(7):467–473. 30178033 10.7326/M18-0850

[35] van Iersel TP , van Spanning SH , Verweij LPE , Priester‐Vink S , van Deurzen DFP , van den Bekerom MPJ . Why do patients with anterior shoulder instability not return to sport after surgery? A systematic review of 63 studies comprising 3545 patients. JSES Int. 2023;7(3):376–384. 37266170 10.1016/j.jseint.2023.01.001 PMC10229421

[36] Verhagen EALM , Van der Beek AJ , Bouter LM , Bahr RM , Mechelen WV . A one season prospective cohort study of volleyball injuries. Br J Sports Med. 2004;38(4):477–481. 15273190 10.1136/bjsm.2003.005785 PMC1724865

[37] Webster KE , Hewett TE . What is the evidence for and validity of return‐to‐sport testing after anterior cruciate ligament reconstruction surgery? A systematic review and meta‐analysis. Sports Med. 2019;49(6):917–929. 30905035 10.1007/s40279-019-01093-x

[38] Williams HLM , Evans JP , Furness ND , Smith CD . It's not all about redislocation: a systematic review of complications after anterior shoulder stabilization surgery. Am J Sports Med. 2019;47(13):3277–3283. 30525905 10.1177/0363546518810711

[39] Wilson KW , Popchak A , Li RT , Kane G , Lin A . Return to sport testing at 6 months after arthroscopic shoulder stabilization reveals residual strength and functional deficits. J Shoulder Elbow Surg. 2020;29(7s):S107–S114. 32643605 10.1016/j.jse.2020.04.035

[40] Yildiz TI , Turhan E , Ocguder DA , Yaman F , Huri G , Duzgun I . Functional performance tests reveal promising results at 6 months after shoulder stabilization surgery. Sports Health. 2023;15(6):878–885. 36539969 10.1177/19417381221141075 PMC10606971

