# Supplementary material for: Objective clinical tests to inform decision‐making prior to return to sport in athletes with shoulder instability: A scoping review
Source: Knee Surg Sports Traumatol Arthrosc. 2025 Oct 27;34(1):268–83. doi: 10.1002/ksa.70107 (PMC12747614; doi:10.1002/ksa.70107)
Supplement: Supplementary file 1 — Supplementary Tables. [file KSA-34-268-s001.docx]

**Supplementary Table 1**

**Online Resource 1**

**Preferred Reporting Items for Systematic reviews and Meta-Analyses extension for Scoping Reviews (PRISMA-ScR) Checklist**

| **SECTION** | **ITEM** | **PRISMA-ScR CHECKLIST ITEM** | **REPORTED ON PAGE #** |
| --- | --- | --- | --- |
| **TITLE** | | | |
| Title | 1 | Identify the report as a scoping review. | 1 |
| **ABSTRACT** | | | |
| Structured summary | 2 | Provide a structured summary that includes (as applicable): background, objectives, eligibility criteria, sources of evidence, charting methods, results, and conclusions that relate to the review questions and objectives. | 3 |
| **INTRODUCTION** | | | |
| Rationale | 3 | Describe the rationale for the review in the context of what is already known. Explain why the review questions/objectives lend themselves to a scoping review approach. | 4 |
| Objectives | 4 | Provide an explicit statement of the questions and objectives being addressed with reference to their key elements (e.g., population or participants, concepts, and context) or other relevant key elements used to conceptualize the review questions and/or objectives. | 4 |
| **METHODS** | | | |
| Protocol and registration | 5 | Indicate whether a review protocol exists; state if and where it can be accessed (e.g., a Web address); and if available, provide registration information, including the registration number. | 5 |
| Eligibility criteria | 6 | Specify characteristics of the sources of evidence used as eligibility criteria (e.g., years considered, language, and publication status), and provide a rationale. | 5 |
| Information sources* | 7 | Describe all information sources in the search (e.g., databases with dates of coverage and contact with authors to identify additional sources), as well as the date the most recent search was executed. | 5 |
| Search | 8 | Present the full electronic search strategy for at least 1 database, including any limits used, such that it could be repeated. | 5-6 |
| Selection of sources of evidence† | 9 | State the process for selecting sources of evidence (i.e., screening and eligibility) included in the scoping review. | 6 |
| Data charting process‡ | 10 | Describe the methods of charting data from the included sources of evidence (e.g., calibrated forms or forms that have been tested by the team before their use, and whether data charting was done independently or in duplicate) and any processes for obtaining and confirming data from investigators. | 6 |
| Data items | 11 | List and define all variables for which data were sought and any assumptions and simplifications made. | 6 |
| Critical appraisal of individual sources of evidence§ | 12 | If done, provide a rationale for conducting a critical appraisal of included sources of evidence; describe the methods used and how this information was used in any data synthesis (if appropriate). | Not applicable |
| Synthesis of results | 13 | Describe the methods of handling and summarizing the data that were charted. | 6 |
| **RESULTS** | | | |
| Selection of sources of evidence | 14 | Give numbers of sources of evidence screened, assessed for eligibility, and included in the review, with reasons for exclusions at each stage, ideally using a flow diagram. | 7 |
| Characteristics of sources of evidence | 15 | For each source of evidence, present characteristics for which data were charted and provide the citations. | 8-9 |
| Critical appraisal within sources of evidence | 16 | If done, present data on critical appraisal of included sources of evidence (see item 12). | Not applicable |
| Results of individual sources of evidence | 17 | For each included source of evidence, present the relevant data that were charted that relate to the review questions and objectives. | 8-17 |
| Synthesis of results | 18 | Summarize and/or present the charting results as they relate to the review questions and objectives. | 7-17 |
| **DISCUSSION** | | | |
| Summary of evidence | 19 | Summarize the main results (including an overview of concepts, themes, and types of evidence available), link to the review questions and objectives, and consider the relevance to key groups. | 18 |
| Limitations | 20 | Discuss the limitations of the scoping review process. | 20 |
| Conclusions | 21 | Provide a general interpretation of the results with respect to the review questions and objectives, as well as potential implications and/or next steps. | 20 |
| **FUNDING** | | | |
| Funding | 22 | Describe sources of funding for the included sources of evidence, as well as sources of funding for the scoping review. Describe the role of the funders of the scoping review. | Not applicable |

JBI = Joanna Briggs Institute; PRISMA-ScR = Preferred Reporting Items for Systematic reviews and Meta-Analyses extension for Scoping Reviews.

* Where *sources of evidence* (see second footnote) are compiled from, such as bibliographic databases, social media platforms, and Web sites.

† A more inclusive/heterogeneous term used to account for the different types of evidence or data sources (e.g., quantitative and/or qualitative research, expert opinion, and policy documents) that may be eligible in a scoping review as opposed to only studies. This is not to be confused with *information sources* (see first footnote).

‡ The frameworks by Arksey and O’Malley (6) and Levac and colleagues (7) and the JBI guidance (4, 5) refer to the process of data extraction in a scoping review as data charting*.*

§ The process of systematically examining research evidence to assess its validity, results, and relevance before using it to inform a decision. This term is used for items 12 and 19 instead of "risk of bias" (which is more applicable to systematic reviews of interventions) to include and acknowledge the various sources of evidence that may be used in a scoping review (e.g., quantitative and/or qualitative research, expert opinion, and policy document).

*From:* Tricco AC, Lillie E, Zarin W, O'Brien KK, Colquhoun H, Levac D, et al. PRISMA Extension for Scoping Reviews (PRISMAScR): Checklist and Explanation. Ann Intern Med. 2018;169:467–473. [doi: 10.7326/M18-0850](http://annals.org/aim/fullarticle/2700389/prisma-extension-scoping-reviews-prisma-scr-checklist-explanation).

**Supplementary Table 2**

**Online Resource 2**

**Database:** MEDLINE (OVID) **Date:** 2022-06-01

**No of results: 1141 ref**

| # | Searches | Results |
| --- | --- | --- |
| 1 | exp Return to Sport/ | 2241 |
| 2 | (Return* or Resumpt* or go back).ab,kf,ti. adj6 (sport* or play* or participat* or compet*).ab,ti,kf. | 11555 |
| 3 | 1 or 2 | 12035 |
| 4 | exp Shoulder Fractures/ or exp Shoulder Pain/ or exp Shoulder Injuries/ or exp Shoulder Joint/ | 38459 |
| 5 | (Shoulder* or Bankart or Hill-Sachs or Glenohumeral or Glenoid Labrum or Superior Labrum from  Anterior to Posterior Injuries or SLAP Tear* or Rotator Cuff or Greater Tuberosit* or Coracohumeral Impingement or Coracoid Impingement or Subacromial Impingement or Outlet Impingement).ab,kf,ti. | 88980 |
| 6 | 4 or 5 | 94479 |
| 7 | 3 and 6 | 1352 |
| 8 | ("Athlet*" or sport* or player* or elite or exercise*).ab,kf,ti. | 507313 |
| 9 | exp Para-Athletes/ or exp Athletes/ or exp Sports/ or exp Athletic Injuries/ | 227559 |
| 10 | 8 or 9 | 617452 |
| 11 | 7 and 10 | 1333 |
| 12 | exp Exercise Test/ | 69555 |
| 13 | (validit* or reliabil* or Isometric* or isokinetic* or overhead* or rate of force development or biodex or dynamometer* or power* or torque total peak or throwing or weak* or pain or instabil* or stabili* or strength or stiffness* or score* or scoring or test* or measur*).ab,kf,ti. | 9288286 |
| 14 | ((closed or open) and chain*).ab,kf,ti. | 17767 |
| 15 | 12 or 13 or 14 | 9316094 |
| 16 | 11 and 15 | 1200 |
| 17 | animals/ not (animals/ and humans/) | 4979391 |
| 18 | (animal or animals or rat or rats or mouse or mice or rodent or rodents or dog or dogs or cat or cats or hamster or hamsters or rabbit or rabbits or swine or murine or porcine or horses or horse).ti. | 2046967 |
| 19 | 17 or 18 | 5364733 |
| 20 | 16 not 19 | 1197 |
| 21 | (comment or editorial or letter).pt. | 2065780 |
| 22 | 20 not 21 | 1176 |
| 23 | limit 22 to (danish or english or norwegian or swedish) | **1141** |

**exp/** = term from the Medline controlled vocabulary, including terms found below this term in the MeSH hierarchy **/** = term from the Medline controlled vocabulary, does not include terms found below this term in the MeSH hierarchy

**.ti,ab,kf**. = title, abstract and author keywords **adjx** = within x words, regardless of order

***** = truncation of word for alternate endings

**Database:** The Cochrane Library

**Date:** 2022-06-08 **No of results: 12 ref**

*Cochrane reviews: 3*

*Cochrane protocols: 0*

*Trials: 9*

*Editorials: 0*

*Special collections: 0*

*Clinical answers: 0*

| ID | Search | Hits |
| --- | --- | --- |
| #1 | MeSH descriptor: [Return to Sport] explode all trees | 50 |
| #2 | ((Return* OR Resumpt* OR go back) NEAR (sport* OR play* OR participat* OR compet*)):ab,ti,kw | 1404 |
| #3 | #1 OR #2 | 1404 |
| #4 | MeSH descriptor: [Shoulder Fractures] explode all trees | 141 |
| #5 | MeSH descriptor: [Shoulder Pain] explode all trees | 1102 |
| #6 | MeSH descriptor: [Shoulder Injuries] explode all trees | 1317 |
| #7 | MeSH descriptor: [Shoulder Joint] explode all trees | 820 |
| #8 | (Shoulder* OR Bankart OR Hill-Sachs OR Glenohumeral OR Glenoid Labrum OR SuperiOR Labrum from Anterior to Posterior Injuries OR SLAP Tear* OR Rotator Cuff OR Greater Tuberosit* OR  Coracohumeral Impingement OR Coracoid Impingement OR Subacromial Impingement OR Outlet Impingement):ti,ab,kw (Word variations have been searched) | 14420 |
| #9 | #4 OR #5 OR #6 OR #7 OR #8 | 14420 |
| #10 | (Athlet* or sport* or player* or elite or exercise*):ti,ab,kw (Word variations have been searched) | 127298 |
| #11 | MeSH descriptor: [Para-Athletes] explode all trees | 1 |
| #12 | MeSH descriptor: [Athletes] explode all trees | 1140 |
| #13 | MeSH descriptor: [Sports] explode all trees | 17130 |
| #14 | MeSH descriptor: [Athletic Injuries] explode all trees | 773 |
| #15 | #10 OR #11 OR #12 OR #13 OR #14 | 130477 |
| #16 | MeSH descriptor: [Exercise Test] explode all trees | 9115 |
| #17 | (validit* OR reliabil* OR Isometric* OR isokinetic* OR overhead* OR rate of force development OR biodex OR dynamometer* OR power* OR torque total peak OR throwing OR weak* OR pain OR instabil* OR stabili* OR strength OR stiffness* OR score* OR scoring OR test* OR measur*):ti,ab,kw (Word variations have been searched) | 993607 |
| #18 | (((closed or open) and chain*)):ti,ab,kw (Word variations have been searched) | 2066 |
| #19 | #16 OR #17 OR #18 | 994185 |
| #20 | #3 AND #9 AND #15 AND 19 | **12** |

**Database:** Embase

**Date:** 2022-06-09

**No of results:** **1186 ref**

| # | Searches | Results |
| --- | --- | --- |
| 1 | exp return to sport/ | 4561 |
| 2 | ((Return* or Resumpt* or go back) adj6 (sport* or play* or participat* or compet*)).ab,kf,ti. | 14036 |
| 3 | 1 or 2 | 15012 |
| 4 | exp shoulder fracture/ | 3839 |
| 5 | exp shoulder pain/ | 19271 |
| 6 | exp shoulder injury/ | 21501 |
| 7 | exp shoulder/ | 76179 |
| 8 | (Shoulder* or Bankart or Hill-Sachs or Glenohumeral or Glenoid Labrum or Superior Labrum from  Anterior to Posterior Injuries or SLAP Tear* or Rotator Cuff or Greater Tuberosit* or  Coracohumeral Impingement or Coracoid Impingement or Subacromial Impingement or Outlet Impingement).ab,kf,ti. | 109340 |
| 9 | 4 or 5 or 6 or 7 or 8 | 146404 |
| 10 | exp disabled athlete/ | 158 |
| 11 | exp athlete/ | 69244 |
| 12 | exp sport/ | 194166 |
| 13 | exp sport injury/ | 32907 |
| 14 | (Athlet* or sport* or player* or elite or exercise*).ab,kf,ti. | 660284 |
| 15 | 10 or 11 or 12 or 13 or 14 | 755041 |
| 16 | exp exercise test/ | 105084 |
| 17 | (validit* or reliabil* or Isometric* or isokinetic* or overhead* or rate of force development or biodex or dynamometer* or power* or torque total peak or throwing or weak* or pain or instabil* or stabili* or strength or stiffness* or score* or scoring or test* or measur*).ab,kf,ti. | 11983455 |
| 18 | ((closed or open) and chain*).ab,kf,ti. | 20042 |
| 19 | 16 or 17 or 18 | 12017529 |
| 20 | 3 and 9 and 15 and 19 | 1509 |
| 21 | animals/ not (animals/ and humans/) | 990611 |
| 22 | (animal or animals or rat or rats or mouse or mice or rodent or rodents or dog or dogs or cat or cats or hamster or hamsters or rabbit or rabbits or swine or murine or porcine or horses or horse).ti. | 2186834 |
| 23 | 21 or 22 | 2943692 |
| 24 | 20 not 21 | 1507 |
| 25 | limit 24 to (books or conference abstract or "conference review" or editorial or erratum or letter or short survey or tombstone) | 272 |
| 26 | 24 not 25 | 1235 |
| 27 | limit 26 to (danish or english or norwegian or swedish) | **1186** |

**Database:** Cinahl **Date:** 2022-06-08

**No of results:** 784 **ref**

| **#** | **Query** | **Results** |
| --- | --- | --- |
| S24 | S20 AND S21 AND S22 AND S23 - Limiters - Language: Danish, English, Norwegian, Swedish  Expanders - Apply related words; Apply equivalent subjects Search modes - Find all my search terms | **784** |
| S23 | S15 OR S16 OR S19 | 1,892,992 |
| S22 | S11 OR S12 OR S13 OR S14 | 259,208 |
| S21 | S5 OR S6 OR S7 OR S8 OR S9 OR S10 | 38,577 |
| S20 | S1 OR S4 | 8,539 |
| S19 | S17 AND S18 | 1,447 |
| S18 | TI chain* OR AB chain* | 53,729 |
| S17 | TI ( closed or open ) OR AB ( closed or open ) | 134,779 |
| S16 | TI ( validit* or reliabil* or Isometric* or isokinetic* or overhead* or rate of force development or biodex or dynamometer* or power* or torque total peak or throwing or weak* or pain or instabil* or stabili* or strength or stiffness* or score* or scoring or test* or measur* ) OR AB ( validit* or reliabil* or Isometric* or isokinetic* or overhead* or rate of force development or biodex or dynamometer* or power* or torque total peak or throwing or weak* or pain or instabil* or stabili* or strength or stiffness* or score* or scoring or test* or measur* ) | 1,884,861 |
| S15 | (MH "Exercise Test+") OR (MH "Exercise Test, Muscular+") | 35,212 |
| S14 | TI ( Athlet* or sport* or player* or elite or exercise* ) OR AB ( Athlet* or sport* or player* or elite or exercise* ) | 214,353 |
| S13 | (MH "Athletic Injuries+") | 24,855 |
| S12 | (MH "Sports+") | 87,349 |
| S11 | (MH "Athletes+") | 32,580 |
| S10 | TI ( Shoulder* or Bankart or Hill-Sachs or Glenohumeral or Glenoid Labrum or Superior Labrum from  Anterior to Posterior Injuries or SLAP Tear* or Rotator Cuff or Greater Tuberosit* or Coracohumeral  Impingement or Coracoid Impingement or Subacromial Impingement or Outlet Impingement ) OR AB  ( Shoulder* or Bankart or Hill-Sachs or Glenohumeral or Glenoid Labrum or Superior Labrum from  Anterior to Posterior Injuries or SLAP Tear* or Rotator Cuff or Greater Tuberosit* or Coracohumeral Impingement or Coracoid Impingement or Subacromial Impingement or Outlet Impingement ) | 35,366 |
| S9 | (MH "Shoulder Labrum Tear") | 196 |
| S8 | (MH "Shoulder Joint+") | 6,885 |
| S7 | (MH "Shoulder Injuries+") | 8,993 |
| S6 | (MH "Shoulder Pain") | 4,471 |
| S5 | (MH "Shoulder Fractures+") | 1,593 |
| S4 | S2 N6 S3 | 7,221 |
| S3 | TI ( sport* or play* or participat* or compet* ) OR AB ( sport* or play* or participat* or compet* ) | 532,388 |
| S2 | TI ( Return* or Resumpt* or go back ) OR AB ( Return* or Resumpt* or go back ) | 73,826 |
| S1 | (MH "Sports Re-Entry") | 3,521 |

**Database:** AMED **Date:** 2022- 06-08

**No of results:** 71 **ref**

| # | Query | Results |
| --- | --- | --- |
| S19 | S15 AND S16 AND S17 AND S18 - Limiters - Language: Danish, English, Norwegian  Expanders - Apply related words; Apply equivalent subjects Search modes - Find all my search terms | **71** |
| S18 | S10 OR S11 OR S14 | 121,764 |
| S17 | S7 OR S8 OR S9 | 31,176 |
| S16 | S5 OR S6 | 5,699 |
| S15 | S1 OR S4 | 1,124 |
| S14 | S12 AND S13 | 275 |
| S13 | TI chain* OR AB chain* | 1,676 |
| S12 | TI ( closed or open ) OR AB ( closed or open ) | 5,982 |
| S11 | TI ( validit* or reliabil* or Isometric* or isokinetic* or overhead* or rate of force development or biodex or dynamometer* or power* or torque total peak or throwing or weak* or pain or instabil* or stabili* or strength or stiffness* or score* or scoring or test* or measur* ) OR AB ( validit* or reliabil* or Isometric* or isokinetic* or overhead* or rate of force development or biodex or dynamometer* or power* or torque total peak or throwing or weak* or pain or instabil* or stabili* or strength or stiffness* or score* or scoring or test* or measur* ) | 120,394 |
| S10 | (ZU "exercise test") or (ZU "exercise testing") or (ZU "exercise tolerance") | 4,641 |
| S9 | TI ( Athlet* or sport* or player* or elite or exercise* ) OR AB ( Athlet* or sport* or player* or elite or exercise* ) | 28,574 |
| S8 | (ZU "sports") or (ZU "sports for persons with disabilities") | 2,806 |
| S7 | (ZU "athletes") or (ZU "athletic injuries") or (ZU "athletics") | 6,826 |
| S6 | TI ( Shoulder* or Bankart or Hill-Sachs or Glenohumeral or Glenoid Labrum or Superior Labrum from Anterior to Posterior Injuries or SLAP Tear* or Rotator Cuff or Greater Tuberosit* or Coracohumeral Impingement or Coracoid Impingement or Subacromial Impingement or Outlet  Impingement ) OR AB ( Shoulder* or Bankart or Hill-Sachs or Glenohumeral or Glenoid Labrum or  Superior Labrum from Anterior to Posterior Injuries or SLAP Tear* or Rotator Cuff or Greater Tuberosit* or Coracohumeral Impingement or Coracoid Impingement or Subacromial Impingement or Outlet Impingement ) | 5,415 |
| S5 | (ZU "shoulder impingement syndrome") or (ZU "shoulder injuries") or (ZU "shoulder joint") or (ZU  "shoulder pain") | 2,357 |
| S4 | S2 N6 S3 | 1,071 |
| S3 | TI ( sport* or play* or participat* or compet* ) OR AB ( sport* or play* or participat* or compet*  ) | 32,374 |
| S2 | TI ( Return* or Resumpt* or go back ) ) OR AB ( Return* or Resumpt* or go back ) ) | 5,992 |
| S1 | (ZU "return to sport") or (ZU "return to sports") | 271 |

**Database:** PEDRO

**Date:** 2022-06-08

**No of results:** **13** **ref**

| Fält |  |
| --- | --- |
| Abstract och Title | Shoulder test |
| Therapy | Fitness training |
| Problem | Pain |
| When searching | Match all search term (AND) |

**Database:** PEDRO

**Date:** 2022-06-08

**No of results:** **59** **ref**

|  |  |
| --- | --- |
| Abstract och Title | Shoulder test |
| Therapy | Strength training |
| Problem | Pain |
| When searching | Match all search term (AND) |

**Supplementary Table 3**

**Online Resource 3**

**Database: MEDLINE (OVID)**

**Date: 2023-05-11**

**No of results: 1264 ref**

| **#** | **Search** | **Results** |
| --- | --- | --- |
| 1 | exp Return to Sport/ | 2471 |
| 2 | (Return* or Resumpt* or go back).ab,kf,ti. adj6 (sport* or play* or participat* or compet*).ab,ti,kf. | 12726 |
| 3 | 1 or 2 | 13213 |
| 4 | exp Shoulder Fractures/ or exp Shoulder Pain/ or exp Shoulder Injuries/ or exp Shoulder Joint/ | 40109 |
| 5 | (Shoulder* or Bankart or Hill-Sachs or Glenohumeral or Glenoid Labrum or Superior Labrum from Anterior to Posterior Injuries or SLAP Tear* or Rotator Cuff or Greater Tuberosit* or Coracohumeral Impingement or Coracoid Impingement or Subacromial Impingement or Outlet Impingement).ab,kf,ti. | 94541 |
| 6 | 4 or 5 | 100116 |
| 7 | 3 and 6 | 1495 |
| 8 | ("Athlet*" or sport* or player* or elite).ab,kf,ti. | 218550 |
| 9 | exp Para-Athletes/ or exp Athletes/ or exp Sports/ or exp Athletic Injuries/ | 236946 |
| 10 | 8 or 9 | 368374 |
| 11 | 7 and 10 | 1474 |
| 12 | exp Exercise Test/ | 70798 |
| 13 | (validit* or reliabil* or Isometric* or isokinetic* or overhead* or rate of force development or biodex or dynamometer* or power* or torque total peak or throwing or weak* or pain or instabil* or stabili* or strength or stiffness* or score* or scoring or test* or measur*).ab,kf,ti. | 9857574 |
| 14 | ((closed or open) and chain*).ab,kf,ti. | 18797 |
| 15 | 12 or 13 or 14 | 9886032 |
| 16 | 11 and 15 | 1326 |
| 17 | animals/ not (animals/ and humans/) | 5085821 |
| 18 | (animal or animals or rat or rats or mouse or mice or rodent or rodents or dog or dogs or cat or cats or hamster or hamsters or rabbit or rabbits or swine or murine or porcine or horses or horse).ti. | 2095673 |
| 19 | 17 or 18 | 5492860 |
| 20 | 16 not 19 | 1322 |
| 21 | (comment or editorial or letter).pt. | 2156906 |
| 22 | 20 not 21 | 1300 |
| 23 | limit 22 to (danish or english or norwegian or swedish) | 1264 |

**exp/ = term from the Medline controlled vocabulary, including terms found below this term in the MeSH hierarchy**

**/ = term from the Medline controlled vocabulary, does not include terms found below this term in the MeSH hierarchy**

**.ti,ab,kf. = title, abstract and author keywords**

**adjx = within x words, regardless of order**

*** = truncation of word for alternate endings**

**Database: The Cochrane Library  
Date: 2023-05-11  
No of results: 13 ref**

***Cochrane reviews: 3* 
*Cochrane protocols: 0*  
*Trials: 10* 
*Editorials: 0* 
*Special collections: 0* 
*Clinical answers: 0***

| **ID** | **Search** | **Hits** |
| --- | --- | --- |
| #1 | MeSH descriptor: [Return to Sport] explode all trees | 70 |
| #2 | ((Return* OR Resumpt* OR go back) NEAR (sport* OR play* OR participat* OR compet*)):ab,ti,kw | 1606 |
| #3 | #1 OR #2 | 1606 |
| #4 | MeSH descriptor: [Shoulder Fractures] explode all trees | 177 |
| #5 | MeSH descriptor: [Shoulder Pain] explode all trees | 1432 |
| #6 | MeSH descriptor: [Shoulder Injuries] explode all trees | 1605 |
| #7 | MeSH descriptor: [Shoulder Joint] explode all trees | 960 |
| #8 | (Shoulder* OR Bankart OR Hill-Sachs OR Glenohumeral OR Glenoid Labrum OR SuperiOR Labrum from Anterior to Posterior Injuries OR SLAP Tear* OR Rotator Cuff OR Greater Tuberosit* OR Coracohumeral Impingement OR Coracoid Impingement OR Subacromial Impingement OR Outlet Impingement):ti,ab,kw (Word variations have been searched) | 16533 |
| #9 | #4 OR #5 OR #6 OR #7 OR #8 | 16533 |
| #10 | (Athlet* or sport* or player* or elite or exercise*):ti,ab,kw (Word variations have been searched) | 143215 |
| #11 | MeSH descriptor: [Para-Athletes] explode all trees | 5 |
| #12 | MeSH descriptor: [Athletes] explode all trees | 1451 |
| #13 | MeSH descriptor: [Sports] explode all trees | 20799 |
| #14 | MeSH descriptor: [Athletic Injuries] explode all trees | 888 |
| #15 | #10 OR #11 OR #12 OR #13 OR #14 | 147393 |
| #16 | MeSH descriptor: [Exercise Test] explode all trees | 10410 |
| #17 | (validit* OR reliabil* OR Isometric* OR isokinetic* OR overhead* OR rate of force development OR biodex OR dynamometer* OR power* OR torque total peak OR throwing OR weak* OR pain OR instabil* OR stabili* OR strength OR stiffness* OR score* OR scoring OR test* OR measur*):ti,ab,kw (Word variations have been searched) | 1091376 |
| #18 | (((closed or open) and chain*)):ti,ab,kw (Word variations have been searched) | 2354 |
| #19 | #16 OR #17 OR #18 | 1092015 |
| #20 | #3 AND #9 AND #15 AND 19 | 13 |

**Database: Embase  
Date: 2023-05-11  
No of results: 1385 ref**

| **#** | **Search** | **Results** |
| --- | --- | --- |
| 1 | exp return to sport/ | 5689 |
| 2 | ((Return* or Resumpt* or go back) adj6 (sport* or play* or participat* or compet*)).ab,kf,ti. | 15777 |
| 3 | 1 or 2 | 16958 |
| 4 | exp shoulder fracture/ | 4502 |
| 5 | exp shoulder pain/ | 20857 |
| 6 | exp shoulder injury/ | 23720 |
| 7 | exp shoulder/ | 86432 |
| 8 | (Shoulder* or Bankart or Hill-Sachs or Glenohumeral or Glenoid Labrum or Superior Labrum from Anterior to Posterior Injuries or SLAP Tear* or Rotator Cuff or Greater Tuberosit* or Coracohumeral Impingement or Coracoid Impingement or Subacromial Impingement or Outlet Impingement).ab,kf,ti. | 118098 |
| 9 | 4 or 5 or 6 or 7 or 8 | 159719 |
| 10 | exp disabled athlete/ | 246 |
| 11 | exp athlete/ | 76920 |
| 12 | exp sport/ | 209840 |
| 13 | exp sport injury/ | 34235 |
| 14 | (Athlet* or sport* or player* or elite or exercise*).ab,kf,ti. | 712213 |
| 15 | 10 or 11 or 12 or 13 or 14 | 814464 |
| 16 | exp exercise test/ | 114382 |
| 17 | (validit* or reliabil* or Isometric* or isokinetic* or overhead* or rate of force development or biodex or dynamometer* or power* or torque total peak or throwing or weak* or pain or instabil* or stabili* or strength or stiffness* or score* or scoring or test* or measur*).ab,kf,ti. | 12930456 |
| 18 | ((closed or open) and chain*).ab,kf,ti. | 21445 |
| 19 | 16 or 17 or 18 | 12966960 |
| 20 | 3 and 9 and 15 and 19 | 1783 |
| 21 | animals/ not (animals/ and humans/) | 1058609 |
| 22 | (animal or animals or rat or rats or mouse or mice or rodent or rodents or dog or dogs or cat or cats or hamster or hamsters or rabbit or rabbits or swine or murine or porcine or horses or horse).ti. | 2272833 |
| 23 | 21 or 22 | 3085041 |
| 24 | 20 not 21 | 1781 |
| 25 | limit 24 to (books or conference abstract or "conference review" or editorial or erratum or letter or short survey or tombstone) | 348 |
| 26 | 24 not 25 | 1433 |
| 27 | limit 26 to (danish or english or norwegian or swedish) | 1382 |

**Database: Cinahl  
Date: 2023-05-11  
No of results: 859 ref**

| **#** | **Query** | **Results** |
| --- | --- | --- |
| S24 | S20 AND S21 AND S22 AND S23 - Limiters - Language: Danish, English, Norwegian, Swedish  Expanders - Apply related words; Apply equivalent subjects  Search modes - Find all my search terms | 859 |
| S23 | S15 OR S16 OR S19 | 1,993,368 |
| S22 | S11 OR S12 OR S13 OR S14 | 269,192 |
| S21 | S5 OR S6 OR S7 OR S8 OR S9 OR S10 | 40,335 |
| S20 | S1 OR S4 | 9,086 |
| S19 | S17 AND S18 | 1,525 |
| S18 | TI chain* OR AB chain* | 56,843 |
| S17 | TI ( closed or open ) OR AB ( closed or open ) | 142,356 |
| S16 | TI ( validit* or reliabil* or Isometric* or isokinetic* or overhead* or rate of force development or biodex or dynamometer* or power* or torque total peak or throwing or weak* or pain or instabil* or stabili* or strength or stiffness* or score* or scoring or test* or measur* ) OR AB ( validit* or reliabil* or Isometric* or isokinetic* or overhead* or rate of force development or biodex or dynamometer* or power* or torque total peak or throwing or weak* or pain or instabil* or stabili* or strength or stiffness* or score* or scoring or test* or measur* ) | 1,985,315 |
| S15 | (MH "Exercise Test+") OR (MH "Exercise Test, Muscular+") | 35,639 |
| S14 | TI ( Athlet* or sport* or player* or elite or exercise* ) OR AB ( Athlet* or sport* or player* or elite or exercise* ) | 226,072 |
| S13 | (MH "Athletic Injuries+") | 24,959 |
| S12 | (MH "Sports+") | 88,183 |
| S11 | (MH "Athletes+") | 34,033 |
| S10 | TI ( Shoulder* or Bankart or Hill-Sachs or Glenohumeral or Glenoid Labrum or Superior Labrum from Anterior to Posterior Injuries or SLAP Tear* or Rotator Cuff or Greater Tuberosit* or Coracohumeral Impingement or Coracoid Impingement or Subacromial Impingement or Outlet Impingement ) OR AB ( Shoulder* or Bankart or Hill-Sachs or Glenohumeral or Glenoid Labrum or Superior Labrum from Anterior to Posterior Injuries or SLAP Tear* or Rotator Cuff or Greater Tuberosit* or Coracohumeral Impingement or Coracoid Impingement or Subacromial Impingement or Outlet Impingement ) | 37,170 |
| S9 | (MH "Shoulder Labrum Tear") | 212 |
| S8 | (MH "Shoulder Joint+") | 7,218 |
| S7 | (MH "Shoulder Injuries+") | 9,267 |
| S6 | (MH "Shoulder Pain") | 4,611 |
| S5 | (MH "Shoulder Fractures+") | 1,673 |
| S4 | S2 N6 S3 | 7,771 |
| S3 | TI ( sport* or play* or participat* or compet* ) OR AB ( sport* or play* or participat* or compet* ) | 565,212 |
| S2 | TI ( Return* or Resumpt* or go back ) OR AB ( Return* or Resumpt* or go back ) | 77,188 |
| S1 | (MH "Sports Re-Entry") | 3,778 |

**Database: AMED  
Date: 2023-05-11  
No of results: 80 ref**

| **#** | **Query** | **Results** |
| --- | --- | --- |
| S19 | S15 AND S16 AND S17 AND S18 - Limiters - Language: Danish, English, Norwegian  Expanders - Apply related words; Apply equivalent subjects  Search modes - Find all my search terms | 80 |
| S18 | S10 OR S11 OR S14 | 126,583 |
| S17 | S7 OR S8 OR S9 | 32,294 |
| S16 | S5 OR S6 | 5,921 |
| S15 | S1 OR S4 | 1,227 |
| S14 | S12 AND S13 | 290 |
| S13 | TI chain* OR AB chain* | 1,809 |
| S12 | TI ( closed or open ) OR AB ( closed or open ) | 6,261 |
| S11 | TI ( validit* or reliabil* or Isometric* or isokinetic* or overhead* or rate of force development or biodex or dynamometer* or power* or torque total peak or throwing or weak* or pain or instabil* or stabili* or strength or stiffness* or score* or scoring or test* or measur* ) OR AB ( validit* or reliabil* or Isometric* or isokinetic* or overhead* or rate of force development or biodex or dynamometer* or power* or torque total peak or throwing or weak* or pain or instabil* or stabili* or strength or stiffness* or score* or scoring or test* or measur* ) | 125,201 |
| S10 | (ZU "exercise test") or (ZU "exercise testing") or (ZU "exercise tolerance") | 4,778 |
| S9 | TI ( Athlet* or sport* or player* or elite or exercise* ) OR AB ( Athlet* or sport* or player* or elite or exercise* ) | 29,671 |
| S8 | (ZU "sports") or (ZU "sports for persons with disabilities") | 2,859 |
| S7 | (ZU "athletes") or (ZU "athletic injuries") or (ZU "athletics") | 7,048 |
| S6 | TI ( Shoulder* or Bankart or Hill-Sachs or Glenohumeral or Glenoid Labrum or Superior Labrum from Anterior to Posterior Injuries or SLAP Tear* or Rotator Cuff or Greater Tuberosit* or Coracohumeral Impingement or Coracoid Impingement or Subacromial Impingement or Outlet Impingement ) OR AB ( Shoulder* or Bankart or Hill-Sachs or Glenohumeral or Glenoid Labrum or Superior Labrum from Anterior to Posterior Injuries or SLAP Tear* or Rotator Cuff or Greater Tuberosit* or Coracohumeral Impingement or Coracoid Impingement or Subacromial Impingement or Outlet Impingement ) | 5,633 |
| S5 | (ZU "shoulder impingement syndrome") or (ZU "shoulder injuries") or (ZU "shoulder joint") or (ZU "shoulder pain") | 2,454 |
| S4 | S2 N6 S3 | 1,170 |
| S3 | TI ( sport* or play* or participat* or compet* ) OR AB ( sport* or play* or participat* or compet* ) | 33,762 |
| S2 | TI ( Return* or Resumpt* or go back ) ) OR AB ( Return* or Resumpt* or go back ) ) | 6,234 |
| S1 | (ZU "return to sport") or (ZU "return to sports") | 338 |

**Database: PEDRO
Date: 2023-05-11  
No of results: 14 ref**

|  |  |
| --- | --- |
| Abstract och Title | Shoulder test |
| Therapy | Fitness training |
| Problem | Pain |
| When searching | Match all search term (AND) |

**Database: PEDRO
Date: 2023-05-11  
No of results: 72 ref**

|  |  |
| --- | --- |
| Abstract och Title | Shoulder test |
| Therapy | Strength training |
| Problem | Pain |
| When searching | Match all search term (AND) |

**Supplementary Table 4**

**Online Resource 4**

**Database: MEDLINE (OVID)**

**Date: 2023-05-11**

**No of results: 1264 ref**

| **#** | **Search** | **Results** |
| --- | --- | --- |
| 1 | exp Return to Sport/ | 2471 |
| 2 | (Return* or Resumpt* or go back).ab,kf,ti. adj6 (sport* or play* or participat* or compet*).ab,ti,kf. | 12726 |
| 3 | 1 or 2 | 13213 |
| 4 | exp Shoulder Fractures/ or exp Shoulder Pain/ or exp Shoulder Injuries/ or exp Shoulder Joint/ | 40109 |
| 5 | (Shoulder* or Bankart or Hill-Sachs or Glenohumeral or Glenoid Labrum or Superior Labrum from Anterior to Posterior Injuries or SLAP Tear* or Rotator Cuff or Greater Tuberosit* or Coracohumeral Impingement or Coracoid Impingement or Subacromial Impingement or Outlet Impingement).ab,kf,ti. | 94541 |
| 6 | 4 or 5 | 100116 |
| 7 | 3 and 6 | 1495 |
| 8 | ("Athlet*" or sport* or player* or elite).ab,kf,ti. | 218550 |
| 9 | exp Para-Athletes/ or exp Athletes/ or exp Sports/ or exp Athletic Injuries/ | 236946 |
| 10 | 8 or 9 | 368374 |
| 11 | 7 and 10 | 1474 |
| 12 | exp Exercise Test/ | 70798 |
| 13 | (validit* or reliabil* or Isometric* or isokinetic* or overhead* or rate of force development or biodex or dynamometer* or power* or torque total peak or throwing or weak* or pain or instabil* or stabili* or strength or stiffness* or score* or scoring or test* or measur*).ab,kf,ti. | 9857574 |
| 14 | ((closed or open) and chain*).ab,kf,ti. | 18797 |
| 15 | 12 or 13 or 14 | 9886032 |
| 16 | 11 and 15 | 1326 |
| 17 | animals/ not (animals/ and humans/) | 5085821 |
| 18 | (animal or animals or rat or rats or mouse or mice or rodent or rodents or dog or dogs or cat or cats or hamster or hamsters or rabbit or rabbits or swine or murine or porcine or horses or horse).ti. | 2095673 |
| 19 | 17 or 18 | 5492860 |
| 20 | 16 not 19 | 1322 |
| 21 | (comment or editorial or letter).pt. | 2156906 |
| 22 | 20 not 21 | 1300 |
| 23 | limit 22 to (danish or english or norwegian or swedish) | 1264 |

**exp/ = term from the Medline controlled vocabulary, including terms found below this term in the MeSH hierarchy**

**/ = term from the Medline controlled vocabulary, does not include terms found below this term in the MeSH hierarchy**

**.ti,ab,kf. = title, abstract and author keywords**

**adjx = within x words, regardless of order**

*** = truncation of word for alternate endings**

**Database: The Cochrane Library  
Date: 2023-05-11  
No of results: 13 ref**

***Cochrane reviews: 3* 
*Cochrane protocols: 0*  
*Trials: 10* 
*Editorials: 0* 
*Special collections: 0* 
*Clinical answers: 0***

| **ID** | **Search** | **Hits** |
| --- | --- | --- |
| #1 | MeSH descriptor: [Return to Sport] explode all trees | 70 |
| #2 | ((Return* OR Resumpt* OR go back) NEAR (sport* OR play* OR participat* OR compet*)):ab,ti,kw | 1606 |
| #3 | #1 OR #2 | 1606 |
| #4 | MeSH descriptor: [Shoulder Fractures] explode all trees | 177 |
| #5 | MeSH descriptor: [Shoulder Pain] explode all trees | 1432 |
| #6 | MeSH descriptor: [Shoulder Injuries] explode all trees | 1605 |
| #7 | MeSH descriptor: [Shoulder Joint] explode all trees | 960 |
| #8 | (Shoulder* OR Bankart OR Hill-Sachs OR Glenohumeral OR Glenoid Labrum OR SuperiOR Labrum from Anterior to Posterior Injuries OR SLAP Tear* OR Rotator Cuff OR Greater Tuberosit* OR Coracohumeral Impingement OR Coracoid Impingement OR Subacromial Impingement OR Outlet Impingement):ti,ab,kw (Word variations have been searched) | 16533 |
| #9 | #4 OR #5 OR #6 OR #7 OR #8 | 16533 |
| #10 | (Athlet* or sport* or player* or elite or exercise*):ti,ab,kw (Word variations have been searched) | 143215 |
| #11 | MeSH descriptor: [Para-Athletes] explode all trees | 5 |
| #12 | MeSH descriptor: [Athletes] explode all trees | 1451 |
| #13 | MeSH descriptor: [Sports] explode all trees | 20799 |
| #14 | MeSH descriptor: [Athletic Injuries] explode all trees | 888 |
| #15 | #10 OR #11 OR #12 OR #13 OR #14 | 147393 |
| #16 | MeSH descriptor: [Exercise Test] explode all trees | 10410 |
| #17 | (validit* OR reliabil* OR Isometric* OR isokinetic* OR overhead* OR rate of force development OR biodex OR dynamometer* OR power* OR torque total peak OR throwing OR weak* OR pain OR instabil* OR stabili* OR strength OR stiffness* OR score* OR scoring OR test* OR measur*):ti,ab,kw (Word variations have been searched) | 1091376 |
| #18 | (((closed or open) and chain*)):ti,ab,kw (Word variations have been searched) | 2354 |
| #19 | #16 OR #17 OR #18 | 1092015 |
| #20 | #3 AND #9 AND #15 AND 19 | 13 |

**Database: Embase  
Date: 2023-05-11  
No of results: 1385 ref**

| **#** | **Search** | **Results** |
| --- | --- | --- |
| 1 | exp return to sport/ | 5689 |
| 2 | ((Return* or Resumpt* or go back) adj6 (sport* or play* or participat* or compet*)).ab,kf,ti. | 15777 |
| 3 | 1 or 2 | 16958 |
| 4 | exp shoulder fracture/ | 4502 |
| 5 | exp shoulder pain/ | 20857 |
| 6 | exp shoulder injury/ | 23720 |
| 7 | exp shoulder/ | 86432 |
| 8 | (Shoulder* or Bankart or Hill-Sachs or Glenohumeral or Glenoid Labrum or Superior Labrum from Anterior to Posterior Injuries or SLAP Tear* or Rotator Cuff or Greater Tuberosit* or Coracohumeral Impingement or Coracoid Impingement or Subacromial Impingement or Outlet Impingement).ab,kf,ti. | 118098 |
| 9 | 4 or 5 or 6 or 7 or 8 | 159719 |
| 10 | exp disabled athlete/ | 246 |
| 11 | exp athlete/ | 76920 |
| 12 | exp sport/ | 209840 |
| 13 | exp sport injury/ | 34235 |
| 14 | (Athlet* or sport* or player* or elite or exercise*).ab,kf,ti. | 712213 |
| 15 | 10 or 11 or 12 or 13 or 14 | 814464 |
| 16 | exp exercise test/ | 114382 |
| 17 | (validit* or reliabil* or Isometric* or isokinetic* or overhead* or rate of force development or biodex or dynamometer* or power* or torque total peak or throwing or weak* or pain or instabil* or stabili* or strength or stiffness* or score* or scoring or test* or measur*).ab,kf,ti. | 12930456 |
| 18 | ((closed or open) and chain*).ab,kf,ti. | 21445 |
| 19 | 16 or 17 or 18 | 12966960 |
| 20 | 3 and 9 and 15 and 19 | 1783 |
| 21 | animals/ not (animals/ and humans/) | 1058609 |
| 22 | (animal or animals or rat or rats or mouse or mice or rodent or rodents or dog or dogs or cat or cats or hamster or hamsters or rabbit or rabbits or swine or murine or porcine or horses or horse).ti. | 2272833 |
| 23 | 21 or 22 | 3085041 |
| 24 | 20 not 21 | 1781 |
| 25 | limit 24 to (books or conference abstract or "conference review" or editorial or erratum or letter or short survey or tombstone) | 348 |
| 26 | 24 not 25 | 1433 |
| 27 | limit 26 to (danish or english or norwegian or swedish) | 1382 |

**Database: Cinahl  
Date: 2023-05-11  
No of results: 859 ref**

| **#** | **Query** | **Results** |
| --- | --- | --- |
| S24 | S20 AND S21 AND S22 AND S23 - Limiters - Language: Danish, English, Norwegian, Swedish  Expanders - Apply related words; Apply equivalent subjects  Search modes - Find all my search terms | 859 |
| S23 | S15 OR S16 OR S19 | 1,993,368 |
| S22 | S11 OR S12 OR S13 OR S14 | 269,192 |
| S21 | S5 OR S6 OR S7 OR S8 OR S9 OR S10 | 40,335 |
| S20 | S1 OR S4 | 9,086 |
| S19 | S17 AND S18 | 1,525 |
| S18 | TI chain* OR AB chain* | 56,843 |
| S17 | TI ( closed or open ) OR AB ( closed or open ) | 142,356 |
| S16 | TI ( validit* or reliabil* or Isometric* or isokinetic* or overhead* or rate of force development or biodex or dynamometer* or power* or torque total peak or throwing or weak* or pain or instabil* or stabili* or strength or stiffness* or score* or scoring or test* or measur* ) OR AB ( validit* or reliabil* or Isometric* or isokinetic* or overhead* or rate of force development or biodex or dynamometer* or power* or torque total peak or throwing or weak* or pain or instabil* or stabili* or strength or stiffness* or score* or scoring or test* or measur* ) | 1,985,315 |
| S15 | (MH "Exercise Test+") OR (MH "Exercise Test, Muscular+") | 35,639 |
| S14 | TI ( Athlet* or sport* or player* or elite or exercise* ) OR AB ( Athlet* or sport* or player* or elite or exercise* ) | 226,072 |
| S13 | (MH "Athletic Injuries+") | 24,959 |
| S12 | (MH "Sports+") | 88,183 |
| S11 | (MH "Athletes+") | 34,033 |
| S10 | TI ( Shoulder* or Bankart or Hill-Sachs or Glenohumeral or Glenoid Labrum or Superior Labrum from Anterior to Posterior Injuries or SLAP Tear* or Rotator Cuff or Greater Tuberosit* or Coracohumeral Impingement or Coracoid Impingement or Subacromial Impingement or Outlet Impingement ) OR AB ( Shoulder* or Bankart or Hill-Sachs or Glenohumeral or Glenoid Labrum or Superior Labrum from Anterior to Posterior Injuries or SLAP Tear* or Rotator Cuff or Greater Tuberosit* or Coracohumeral Impingement or Coracoid Impingement or Subacromial Impingement or Outlet Impingement ) | 37,170 |
| S9 | (MH "Shoulder Labrum Tear") | 212 |
| S8 | (MH "Shoulder Joint+") | 7,218 |
| S7 | (MH "Shoulder Injuries+") | 9,267 |
| S6 | (MH "Shoulder Pain") | 4,611 |
| S5 | (MH "Shoulder Fractures+") | 1,673 |
| S4 | S2 N6 S3 | 7,771 |
| S3 | TI ( sport* or play* or participat* or compet* ) OR AB ( sport* or play* or participat* or compet* ) | 565,212 |
| S2 | TI ( Return* or Resumpt* or go back ) OR AB ( Return* or Resumpt* or go back ) | 77,188 |
| S1 | (MH "Sports Re-Entry") | 3,778 |

**Database: AMED  
Date: 2023-05-11  
No of results: 80 ref**

| # | **Query** | **Results** |
| --- | --- | --- |
| S19 | S15 AND S16 AND S17 AND S18 - Limiters - Language: Danish, English, Norwegian  Expanders - Apply related words; Apply equivalent subjects  Search modes - Find all my search terms | 80 |
| S18 | S10 OR S11 OR S14 | 126,583 |
| S17 | S7 OR S8 OR S9 | 32,294 |
| S16 | S5 OR S6 | 5,921 |
| S15 | S1 OR S4 | 1,227 |
| S14 | S12 AND S13 | 290 |
| S13 | TI chain* OR AB chain* | 1,809 |
| S12 | TI ( closed or open ) OR AB ( closed or open ) | 6,261 |
| S11 | TI ( validit* or reliabil* or Isometric* or isokinetic* or overhead* or rate of force development or biodex or dynamometer* or power* or torque total peak or throwing or weak* or pain or instabil* or stabili* or strength or stiffness* or score* or scoring or test* or measur* ) OR AB ( validit* or reliabil* or Isometric* or isokinetic* or overhead* or rate of force development or biodex or dynamometer* or power* or torque total peak or throwing or weak* or pain or instabil* or stabili* or strength or stiffness* or score* or scoring or test* or measur* ) | 125,201 |
| S10 | (ZU "exercise test") or (ZU "exercise testing") or (ZU "exercise tolerance") | 4,778 |
| S9 | TI ( Athlet* or sport* or player* or elite or exercise* ) OR AB ( Athlet* or sport* or player* or elite or exercise* ) | 29,671 |
| S8 | (ZU "sports") or (ZU "sports for persons with disabilities") | 2,859 |
| S7 | (ZU "athletes") or (ZU "athletic injuries") or (ZU "athletics") | 7,048 |
| S6 | TI ( Shoulder* or Bankart or Hill-Sachs or Glenohumeral or Glenoid Labrum or Superior Labrum from Anterior to Posterior Injuries or SLAP Tear* or Rotator Cuff or Greater Tuberosit* or Coracohumeral Impingement or Coracoid Impingement or Subacromial Impingement or Outlet Impingement ) OR AB ( Shoulder* or Bankart or Hill-Sachs or Glenohumeral or Glenoid Labrum or Superior Labrum from Anterior to Posterior Injuries or SLAP Tear* or Rotator Cuff or Greater Tuberosit* or Coracohumeral Impingement or Coracoid Impingement or Subacromial Impingement or Outlet Impingement ) | 5,633 |
| S5 | (ZU "shoulder impingement syndrome") or (ZU "shoulder injuries") or (ZU "shoulder joint") or (ZU "shoulder pain") | 2,454 |
| S4 | S2 N6 S3 | 1,170 |
| S3 | TI ( sport* or play* or participat* or compet* ) OR AB ( sport* or play* or participat* or compet* ) | 33,762 |
| S2 | TI ( Return* or Resumpt* or go back ) ) OR AB ( Return* or Resumpt* or go back ) ) | 6,234 |
| S1 | (ZU "return to sport") or (ZU "return to sports") | 338 |

**Database: PEDRO
Date: 2023-05-11  
No of results: 14 ref**

|  |  |
| --- | --- |
| Abstract och Title | Shoulder test |
| Therapy | Fitness training |
| Problem | Pain |
| When searching | Match all search term (AND) |

**Database: PEDRO – sökning på Strength training 
Date: 2023-05-11  
No of results: 72 ref**

|  |  |
| --- | --- |
| Abstract och Title | Shoulder test |
| Therapy | Strength training |
| Problem | Pain |
| When searching | Match all search term (AND) |

**Database:** MEDLINE (OVID)

**Date:** 2023-05-11

**No of results:** 1264 ref

| **#** | **Search** | **Results** |
| --- | --- | --- |
| 1 | exp Return to Sport/ | 2471 |
| 2 | (Return* or Resumpt* or go back).ab,kf,ti. adj6 (sport* or play* or participat* or compet*).ab,ti,kf. | 12726 |
| 3 | 1 or 2 | 13213 |
| 4 | exp Shoulder Fractures/ or exp Shoulder Pain/ or exp Shoulder Injuries/ or exp Shoulder Joint/ | 40109 |
| 5 | (Shoulder* or Bankart or Hill-Sachs or Glenohumeral or Glenoid Labrum or Superior Labrum from Anterior to Posterior Injuries or SLAP Tear* or Rotator Cuff or Greater Tuberosit* or Coracohumeral Impingement or Coracoid Impingement or Subacromial Impingement or Outlet Impingement).ab,kf,ti. | 94541 |
| 6 | 4 or 5 | 100116 |
| 7 | 3 and 6 | 1495 |
| 8 | ("Athlet*" or sport* or player* or elite).ab,kf,ti. | 218550 |
| 9 | exp Para-Athletes/ or exp Athletes/ or exp Sports/ or exp Athletic Injuries/ | 236946 |
| 10 | 8 or 9 | 368374 |
| 11 | 7 and 10 | 1474 |
| 12 | exp Exercise Test/ | 70798 |
| 13 | (validit* or reliabil* or Isometric* or isokinetic* or overhead* or rate of force development or biodex or dynamometer* or power* or torque total peak or throwing or weak* or pain or instabil* or stabili* or strength or stiffness* or score* or scoring or test* or measur*).ab,kf,ti. | 9857574 |
| 14 | ((closed or open) and chain*).ab,kf,ti. | 18797 |
| 15 | 12 or 13 or 14 | 9886032 |
| 16 | 11 and 15 | 1326 |
| 17 | animals/ not (animals/ and humans/) | 5085821 |
| 18 | (animal or animals or rat or rats or mouse or mice or rodent or rodents or dog or dogs or cat or cats or hamster or hamsters or rabbit or rabbits or swine or murine or porcine or horses or horse).ti. | 2095673 |
| 19 | 17 or 18 | 5492860 |
| 20 | 16 not 19 | 1322 |
| 21 | (comment or editorial or letter).pt. | 2156906 |
| 22 | 20 not 21 | 1300 |
| 23 | limit 22 to (danish or english or norwegian or swedish) | 1264 |

**exp/** = term from the Medline controlled vocabulary, including terms found below this term in the MeSH hierarchy

**/** = term from the Medline controlled vocabulary, does not include terms found below this term in the MeSH hierarchy

**.ti,ab,kf**. = title, abstract and author keywords

**adjx** = within x words, regardless of order

***** = truncation of word for alternate endings

**Database:** The Cochrane Library
**Date:** 2023-05-11
**No of results:** 13 ref

*Cochrane reviews: 3
Cochrane protocols: 0
Trials: 10
Editorials: 0
Special collections: 0
Clinical answers: 0*

| **ID** | **Search** | **Hits** |
| --- | --- | --- |
| #1 | MeSH descriptor: [Return to Sport] explode all trees | 70 |
| #2 | ((Return* OR Resumpt* OR go back) NEAR (sport* OR play* OR participat* OR compet*)):ab,ti,kw | 1606 |
| #3 | #1 OR #2 | 1606 |
| #4 | MeSH descriptor: [Shoulder Fractures] explode all trees | 177 |
| #5 | MeSH descriptor: [Shoulder Pain] explode all trees | 1432 |
| #6 | MeSH descriptor: [Shoulder Injuries] explode all trees | 1605 |
| #7 | MeSH descriptor: [Shoulder Joint] explode all trees | 960 |
| #8 | (Shoulder* OR Bankart OR Hill-Sachs OR Glenohumeral OR Glenoid Labrum OR SuperiOR Labrum from Anterior to Posterior Injuries OR SLAP Tear* OR Rotator Cuff OR Greater Tuberosit* OR Coracohumeral Impingement OR Coracoid Impingement OR Subacromial Impingement OR Outlet Impingement):ti,ab,kw (Word variations have been searched) | 16533 |
| #9 | #4 OR #5 OR #6 OR #7 OR #8 | 16533 |
| #10 | (Athlet* or sport* or player* or elite or exercise*):ti,ab,kw (Word variations have been searched) | 143215 |
| #11 | MeSH descriptor: [Para-Athletes] explode all trees | 5 |
| #12 | MeSH descriptor: [Athletes] explode all trees | 1451 |
| #13 | MeSH descriptor: [Sports] explode all trees | 20799 |
| #14 | MeSH descriptor: [Athletic Injuries] explode all trees | 888 |
| #15 | #10 OR #11 OR #12 OR #13 OR #14 | 147393 |
| #16 | MeSH descriptor: [Exercise Test] explode all trees | 10410 |
| #17 | (validit* OR reliabil* OR Isometric* OR isokinetic* OR overhead* OR rate of force development OR biodex OR dynamometer* OR power* OR torque total peak OR throwing OR weak* OR pain OR instabil* OR stabili* OR strength OR stiffness* OR score* OR scoring OR test* OR measur*):ti,ab,kw (Word variations have been searched) | 1091376 |
| #18 | (((closed or open) and chain*)):ti,ab,kw (Word variations have been searched) | 2354 |
| #19 | #16 OR #17 OR #18 | 1092015 |
| #20 | #3 AND #9 AND #15 AND 19 | **13** |

**Supplementary Table 5**

**Online Resource 5**

**Database:** Embase
**Date:** 2025-07-30
**No of results:** 1658 ref

| **#** | **Search** | **Results** |
| --- | --- | --- |
| 1 | exp return to sport/ | 8275 |
| 2 | ((Return* or Resumpt* or go back) adj6 (sport* or play* or participat* or compet*)).ab,kf,ti. | 19517 |
| 3 | 1 or 2 | 21191 |
| 4 | exp shoulder fracture/ | 6363 |
| 5 | exp shoulder pain/ | 25379 |
| 6 | exp shoulder injury/ | 30358 |
| 7 | exp shoulder/ | 100696 |
| 8 | (Shoulder* or Bankart or Hill-Sachs or Glenohumeral or Glenoid Labrum or Superior Labrum from Anterior to Posterior Injuries or SLAP Tear* or Rotator Cuff or Greater Tuberosit* or Coracohumeral Impingement or Coracoid Impingement or Subacromial Impingement or Outlet Impingement).ab,kf,ti. | 140249 |
| 9 | 4 or 5 or 6 or 7 or 8 | 189555 |
| 10 | exp disabled athlete/ | 462 |
| 11 | exp athlete/ | 92236 |
| 12 | exp sport/ | 245534 |
| 13 | exp sport injury/ | 39888 |
| 14 | (Athlet* or sport* or player* or elite or exercise*).ab,kf,ti. | 841581 |
| 15 | 10 or 11 or 12 or 13 or 14 | 959099 |
| 16 | exp exercise test/ | 139724 |
| 17 | (validit* or reliabil* or Isometric* or isokinetic* or overhead* or rate of force development or biodex or dynamometer* or power* or torque total peak or throwing or weak* or pain or instabil* or stabili* or strength or stiffness* or score* or scoring or test* or measur*).ab,kf,ti. | 14811044 |
| 18 | ((closed or open) and chain*).ab,kf,ti. | 24907 |
| 19 | 16 or 17 or 18 | 14852971 |
| 20 | 3 and 9 and 15 and 19 | 2297 |
| 21 | animals/ not (animals/ and humans/) | 1261027 |
| 22 | (animal or animals or rat or rats or mouse or mice or rodent or rodents or dog or dogs or cat or cats or hamster or hamsters or rabbit or rabbits or swine or murine or porcine or horses or horse).ti. | 2399293 |
| 23 | 21 or 22 | 3374862 |
| 24 | 20 not 21 | 2295 |
| 25 | limit 24 to (books or conference abstract or "conference review" or editorial or erratum or letter or short survey or tombstone) | 508 |
| 26 | 24 not 25 | 1787 |
| 27 | limit 26 to (danish or english or norwegian or swedish) | 1737 |
| 28 | limit 27 to clinical trial | 79 |
| 29 | 27 not 28 | 1658 |

**Database:** Cinahl
**Date:** 2023-05-11
**No of results:** 859 ref

| **#** | **Query** | **Results** |
| --- | --- | --- |
| S24 | S20 AND S21 AND S22 AND S23 - Limiters - Language: Danish, English, Norwegian, Swedish Expanders - Apply related words; Apply equivalent subjects Search modes - Find all my search terms | **859** |
| S23 | S15 OR S16 OR S19 | 1,993,368 |
| S22 | S11 OR S12 OR S13 OR S14 | 269,192 |
| S21 | S5 OR S6 OR S7 OR S8 OR S9 OR S10 | 40,335 |
| S20 | S1 OR S4 | 9,086 |
| S19 | S17 AND S18 | 1,525 |
| S18 | TI chain* OR AB chain* | 56,843 |
| S17 | TI ( closed or open ) OR AB ( closed or open ) | 142,356 |
| S16 | TI ( validit* or reliabil* or Isometric* or isokinetic* or overhead* or rate of force development or biodex or dynamometer* or power* or torque total peak or throwing or weak* or pain or instabil* or stabili* or strength or stiffness* or score* or scoring or test* or measur* ) OR AB ( validit* or reliabil* or Isometric* or isokinetic* or overhead* or rate of force development or biodex or dynamometer* or power* or torque total peak or throwing or weak* or pain or instabil* or stabili* or strength or stiffness* or score* or scoring or test* or measur* ) | 1,985,315 |
| S15 | (MH "Exercise Test+") OR (MH "Exercise Test, Muscular+") | 35,639 |
| S14 | TI ( Athlet* or sport* or player* or elite or exercise* ) OR AB ( Athlet* or sport* or player* or elite or exercise* ) | 226,072 |
| S13 | (MH "Athletic Injuries+") | 24,959 |
| S12 | (MH "Sports+") | 88,183 |
| S11 | (MH "Athletes+") | 34,033 |
| S10 | TI ( Shoulder* or Bankart or Hill-Sachs or Glenohumeral or Glenoid Labrum or Superior Labrum from Anterior to Posterior Injuries or SLAP Tear* or Rotator Cuff or Greater Tuberosit* or Coracohumeral Impingement or Coracoid Impingement or Subacromial Impingement or Outlet Impingement ) OR AB ( Shoulder* or Bankart or Hill-Sachs or Glenohumeral or Glenoid Labrum or Superior Labrum from Anterior to Posterior Injuries or SLAP Tear* or Rotator Cuff or Greater Tuberosit* or Coracohumeral Impingement or Coracoid Impingement or Subacromial Impingement or Outlet Impingement ) | 37,170 |
| S9 | (MH "Shoulder Labrum Tear") | 212 |
| S8 | (MH "Shoulder Joint+") | 7,218 |
| S7 | (MH "Shoulder Injuries+") | 9,267 |
| S6 | (MH "Shoulder Pain") | 4,611 |
| S5 | (MH "Shoulder Fractures+") | 1,673 |
| S4 | S2 N6 S3 | 7,771 |
| S3 | TI ( sport* or play* or participat* or compet* ) OR AB ( sport* or play* or participat* or compet* ) | 565,212 |
| S2 | TI ( Return* or Resumpt* or go back ) OR AB ( Return* or Resumpt* or go back ) | 77,188 |
| S1 | (MH "Sports Re-Entry") | 3,778 |

Formulärets nederkant

**Database:** AMED
**Date:** 2023-05-11
**No of results:** 80 ref

| **#** | **Query** | **Results** |
| --- | --- | --- |
| S19 | S15 AND S16 AND S17 AND S18 - Limiters - Language: Danish, English, Norwegian Expanders - Apply related words; Apply equivalent subjects Search modes - Find all my search terms | **80** |
| S18 | S10 OR S11 OR S14 | 126,583 |
| S17 | S7 OR S8 OR S9 | 32,294 |
| S16 | S5 OR S6 | 5,921 |
| S15 | S1 OR S4 | 1,227 |
| S14 | S12 AND S13 | 290 |
| S13 | TI chain* OR AB chain* | 1,809 |
| S12 | TI ( closed or open ) OR AB ( closed or open ) | 6,261 |
| S11 | TI ( validit* or reliabil* or Isometric* or isokinetic* or overhead* or rate of force development or biodex or dynamometer* or power* or torque total peak or throwing or weak* or pain or instabil* or stabili* or strength or stiffness* or score* or scoring or test* or measur* ) OR AB ( validit* or reliabil* or Isometric* or isokinetic* or overhead* or rate of force development or biodex or dynamometer* or power* or torque total peak or throwing or weak* or pain or instabil* or stabili* or strength or stiffness* or score* or scoring or test* or measur* ) | 125,201 |
| S10 | (ZU "exercise test") or (ZU "exercise testing") or (ZU "exercise tolerance") | 4,778 |
| S9 | TI ( Athlet* or sport* or player* or elite or exercise* ) OR AB ( Athlet* or sport* or player* or elite or exercise* ) | 29,671 |
| S8 | (ZU "sports") or (ZU "sports for persons with disabilities") | 2,859 |
| S7 | (ZU "athletes") or (ZU "athletic injuries") or (ZU "athletics") | 7,048 |
| S6 | TI ( Shoulder* or Bankart or Hill-Sachs or Glenohumeral or Glenoid Labrum or Superior Labrum from Anterior to Posterior Injuries or SLAP Tear* or Rotator Cuff or Greater Tuberosit* or Coracohumeral Impingement or Coracoid Impingement or Subacromial Impingement or Outlet Impingement ) OR AB ( Shoulder* or Bankart or Hill-Sachs or Glenohumeral or Glenoid Labrum or Superior Labrum from Anterior to Posterior Injuries or SLAP Tear* or Rotator Cuff or Greater Tuberosit* or Coracohumeral Impingement or Coracoid Impingement or Subacromial Impingement or Outlet Impingement ) | 5,633 |
| S5 | (ZU "shoulder impingement syndrome") or (ZU "shoulder injuries") or (ZU "shoulder joint") or (ZU "shoulder pain") | 2,454 |
| S4 | S2 N6 S3 | 1,170 |
| S3 | TI ( sport* or play* or participat* or compet* ) OR AB ( sport* or play* or participat* or compet* ) | 33,762 |
| S2 | TI ( Return* or Resumpt* or go back ) ) OR AB ( Return* or Resumpt* or go back ) ) | 6,234 |
| S1 | (ZU "return to sport") or (ZU "return to sports") | 338 |

Formulärets nederkant

**Database:** PEDRO
**Date:** 2025-07-30
**No of results:** **16** ref

| Fält |  |
| --- | --- |
| Abstract och Title | Shoulder test |
| Therapy | Fitness training |
| Problem | Pain |
| When searching | Match all search term (AND) |

**Database:** PEDRO
**Date:** 2025-07-30
**No of results:** **95** ref

| Fält |  |
| --- | --- |
| Abstract och Title | Shoulder test |
| Therapy | Strength training |
| Problem | Pain |
| When searching | Match all search term (AND) |
